# Peptidomimetic inhibitors of the VEGF-A_165_/NRP-1 complex obtained by modification of the C-terminal arginine

**DOI:** 10.1007/s00726-024-03411-8

**Published:** 2024-08-24

**Authors:** Dagmara Tymecka, Patrycja Redkiewicz, Piotr F. J. Lipiński, Aleksandra Misicka

**Affiliations:** 1 Faculty of Chemistry, University of Warsaw, Pasteura 1, 02-093 Warsaw, Poland; 2 Department of Neuropeptides, Mossakowski Medical Research Institute Polish Academy of Sciences, Pawińskiego 5, 02-106 Warsaw, Poland

**Keywords:** VEGF_165_/NRP-1 complex inhibitor, Peptidomimetics, Branched peptide, SAR, Arginine analogues, Neuropilin-1

## Abstract

Inhibitors of the interaction between Neuropilin-1 (NRP-1) and Vascular Endothelial Growth Factor-A_165_ (VEGF-A_165_) hold significant promise as therapeutic and diagnostic agents directed against cancers overexpressing NRP-1. In our efforts in this field, a few series of strong and fairly stable peptide-like inhibitors of the general formula Lys(Har)^1^-Xaa^2^-Xaa^3^-Arg^4^ have been previously discovered. In the current work, we focused on Lys(Har)-Dap/Dab-Pro-Arg sequence. The aim was to examine whether replacing C-terminal Arg with its homologs and mimetics would yield more stable yet still potent inhibitors. Upon considering the results of modelling and other factors, ten novel analogues with Xaa^4^ = homoarginine (Har), 2-amino-4-guanidino-butyric acid (Agb), 2-amino-3-guanidino-propionic acid (Agp), citrulline (Cit), 4-aminomethyl-phenylalanine [Phe(4-CH_2_-NH_2_)] were designed, synthesized and evaluated. Two of the proposed modifications resulted in inhibitors with activity slightly lower [e.g. IC_50_ = 14.3 μM for Lys(Har)-Dab-Pro-Har and IC_50_ = 19.8 μM for Lys(Har)-Dab-Pro-Phe(4-CH_2_-NH_2_)] than the parent compounds [e.g. IC_50_ = 4.7 μM for Lys(Har)-Dab-Pro-Arg]. What was a surprise to us, the proteolytic stability depended more on position two of the sequence than on position four. The Dab^2^-analogues exhibited half-life times beyond 60 h. Our results build up the knowledge on the structural requirements that effective VEGF-A_165_/NRP-1 inhibitors should fulfil.

**Supplementary Information:**

The online version contains supplementary material available at 10.1007/s00726-024-03411-8.

## Introduction

Neuropilin-1 (NRP-1) is a cell surface receptor involved, inter alia, in the development of axon guidance and also in physiological, as well as pathological angiogenesis processes, including cancer (He and Tessier-Lavigne 1997; Lee et al. 2002; Staton et al. 2007). This single-pass transmembrane glycoprotein plays a crucial role in the formation of capillaries in the angiogenesis process. Its overexpression is associated with tumor aggressiveness and metastasis as observed e.g. in breast (Stephenson et al. 2002), pancreas (Parikh et al. 2003), prostate (Latil et al. 2000) or colon (Parikh et al. 2004) cancers. One of the most important ligands of NRP-1 and the main mediators of angiogenesis processes is the vascular endothelial growth factor-A_165_ (VEGF-A_165_), which acts as a proangiogenic factor that interacts with b1 and b2 of the NRP-1 subdomains. Compounds that block this interaction are potential inhibitors of the VEGF-A_165_/NRP-1 complex that may find application in the diagnosis and therapy of cancer (Peng et al. 2019). NRP-1 is investigated as a promising target for the targeted delivery of diagnostic or therapeutic radionuclides (Adhikari et al. 2019; Moussaron et al. 2021; Masłowska et al. 2022, 2023). Outside of the oncological field, recent data point to the possibility of using VEGF-A_165_/NRP-1 inhibitors as anti-SARS-CoV-2 agents (Cantuti-Castelvetri et al. 2020; Daly et al. 2020; Hu et al. 2023) or as a novel treatment strategy for neuropathic pain (Stratton et al. 2023).

The so far proposed inhibitors of the VEGF-A_165_/NRP-1 complex include cyclic (Jia et al. 2006, 2014; Grabowska et al. 2016, 2017) or linear (von Wronski et al. 2006; Starzec et al. 2006, 2007; Vander Kooi et al. 2007; Kamarulzaman et al. 2017; Fedorczyk et al. 2017; Tymecka et al. 2017, 2018; Puszko et al. 2019, 2020, 2023) peptides/peptidomimetics and small molecules (Jarvis et al. 2010; Novoa et al. 2010; Borriello et al. 2014; Starzec et al. 2014; Liu et al. 2014, 2018; Richard et al. 2016; Powell et al. 2018). In the group of small molecules, the significant result was the design of EG00229 that has been shown by in vivo assays to be a promising anticancer compound (Jarvis et al. 2010). Another small molecular inhibitor with demonstrated in vivo anticancer activity is NRPa-308 (Liu et al. 2018). In the peptides group, the significant achievement was the identification (by a mutated phage library screening) of heptapeptide Ala-Thr-Trp-Leu-Pro-Pro-Arg (A7R) which selectively inhibits VEGF-A_165_ binding to NRP-1 and decreases breast cancer angiogenesis and growth in vivo (Starzec et al. 2006, 2007).

Structure–Activity Relationship (SAR) studies around A7R showed that the shortest active fragment is its C-terminal tetrapeptide Leu-Pro-Pro-Arg. In the course of further studies, we found that substitution of Leu in the first position by Lys, and additionally the extension of the side chain of Lys by attachment of homoarginine (Har) residue, provides more active and more stable analogues. Moreover, increasing the flexibility of the middle part of molecule, in particular with simultaneous introduction of additional receptor interacting elements, at the second or third position, produced branched pentapeptides up to 30-fold more active than the A7R (Tymecka et al. 2018).

Another key SAR finding, relevant to all peptidic inhibitors of the VEGF-A_165_/NRP-1 interactions, is that the C-terminal arginine is crucial for binding to the NRP-1 and its lack is associated with a drastic decrease or even loss of inhibitory activity (Starzec et al. 2007; Jarvis et al. 2010). On the other hand, the metabolic stability studies of our branched pentapeptides Lys(Har)^1^-Xaa^2^-Xaa^3^-Arg^4^ have shown that among the first cleavage sites is the detachment of this key arginine (Tymecka et al. 2018; Puszko et al. 2023).

In view of the above information, we wondered whether exchange of C-terminal Arg for its homologs and mimetics in our two previously presented compounds, Lys(Har)-Dap-Pro-Arg (**1**) and Lys(Har)-Dab-Pro-Arg (**2**), would yield more stable yet still potent inhibitors. This problem was of practical interest in our works towards optimization of these analogues. It is a common knowledge that insufficient stability of peptide active substances is their major liability, significantly hampering their practical applications. Our inhibitors **1** and **2** displayed good stabilities with t_½_’s beyond 24 h (Tymecka et al. 2018), that nonetheless were deemed desirable to improve. In this paper we report on design, synthesis and serum metabolic stability of several C-terminally modified analogues (compounds **3**–**12**) of our leads.

## Materials and methods

### Molecular modelling

Compounds contemplated to be synthesized, including analogues **3**–**12**, were modelled in the NRP-1 binding site by manual docking followed with scoring using AutoDock Vina (Trott and Olson 2010). The basis for the modelling were the 2ORZ structure (Vander Kooi et al. 2007) from the PDB database (Yin et al. 2018) and two snapshot structures from our molecular dynamics (MD) simulations performed for compound **2** bound to NRP-1 (started also from the 2ORZ structure), described previously (Tymecka et al. 2018). The frames were representative of two binding poses (BP1 and BP2) that were significantly sampled during the MD runs of NRP-1 bound with our branched pentapeptide inhibitors.

The C-terminally modified analogues of **1** and **2** were sculpted manually (by modifying the structure of **2** from the MD snapshots) in the BP1 or BP2 binding orientation using the Discovery Studio (Biovia Discovery Studio Visualizer 2018). The manually prepared conformations were minimized (cleaned), followed by minimization of protein hydrogens. Alternatively, model fragments Ac-Pro-Xaa-OH (where Xaa stands for the contemplated C-terminal residue) were similarly prepared in the NRP-1 binding site. After the preparations, the ligands and proteins structures were converted to suitable input files using AutoDock Tools (Morris et al. 2009). The obtained complexes were scored with AutoDock Vina (Trott and Olson 2010) (using the *score_only* option, thus omitting the search procedure).

In all dockings, the protonation states for both ligands and the protein were set as expected at pH 7.4. The post-modelling analyses included visual inspection (Fischer et al. 2021) and comparison of the designs’ scores to those of the parent compounds. Molecular graphics were prepared in Pymol (Schrödinger LLC 2018) and Chimera (Pettersen et al. 2004).

### Peptidomimetic synthesis

Unless otherwise specified, reagents and solvents were obtained from commercial suppliers and used without further purification. Fmoc-Arg(Pbf) Wang resin, Fmoc-Har(Pbf)-OH, Fmoc-Cit-OH and 4-(Boc-aminomethyl)-N-Fmoc-l-phenylalanine were purchased from Merck (Poland). 2-Chlorotrityl chloride resin, other amino acids, coupling reagents (DIC/Oxyma) and solvents were purchased from Iris Biotech (Marktredwitz, Germany). The Oxy-B and TBEC reagent were given by Luxembourg Bio Technologies LTD (Israel).

The designed compounds were synthesized by a standard 9-fluorenylmethoxycarbonyl (Fmoc) solid phase peptide synthesis methodology as previously reported (Fedorczyk et al. 2017). Preloaded Fmoc-Arg(Pbf)-Wang resin was used for the synthesis of parent compounds (**1**–**2**). The synthesis of all other peptidomimetics **3**–**12** was carried out on 2-Chlorotrityl chloride resin, using standard loading protocol for attachment of the first amino acid to the resin (Fmoc-Xaa-OH: DIPEA 0.6 eq./4 eq. in dry DCM). Final compounds were cleaved from the resin with the use of cocktail TFA:phenol:H_2_O:TIS (88:5:5:2, v/v). The crude peptidomimetics were purified by reversed phase high performance liquid chromatography (RP-HPLC), then analysed with liquid chromatography coupled with mass spectrometry (LC–MS), (Supplemental Figure SI-25 and Supplemental Table SI-3). The structure of each peptide was confirmed by high resolution mass spectrometry (HR-MS), (Supplemental Table SI-2).

### Inhibition of VEGF-A_165_/NRP-1 binding assay

Inhibitory activity of the synthesized peptides was measured by a competitive ELISA test, following a protocol similar to the one previously described (Puszko et al. 2019). Briefly, the first step was to coat the bottom surface of a 96-well plate using 100 µl (200 ng/well) of recombinant human NRP-1 (BioLegend, San Diego, CA, USA) and incubate overnight at 4 °C. Nonspecific binding was blocked with 0.5% BSA (BioShop, Canada) in PBS (Bioshop, Canada). Then, 50 µl peptide in PBS in the selected concentrations and 50 µl (400 ng/ml) human (bt)-(bt) VEGF-A_165_ (Abcam, Cambridge, UK) in PBS containing 4 µg/ml heparin (Bioshop, Canada) were added. After 2 h of incubation at RT, the plate was washed and treated with ECL Streptavidin–Horseradish Peroxidase conjugate (GE Healthcare, Little Chalfont, UK) in PBS (1:100) for 45 min. After the final wash the chemiluminescence was quantified immediately after addition of 100 μl chemiluminescent substrate (SuperSignal ELISA Pico Chemiluminescent Substrate; Pierce Biotechnology, Rockford, IL, USA). Wells incubated with (bt)-VEGF-A_165_ served as a positive control (P), while wells not-coated with NRP-1 were used as a negative control (NS). Percentages of inhibition were calculated according to the following formula:

$${1}00\% \, - \, \left[ {\left[ {\left( {{\text{S }} - {\text{ NS}}} \right)/\left( {{\text{P }} - {\text{ NS}}} \right)} \right] \, \times { 1}00\% } \right]$$

where S is the signal intensity obtained from wells incubated with the studied compound. Half-maximal inhibitory concentration (IC_50_, [µM]) values (mean ± SD of three independent experiments were determined by nonlinear regression analysis (log(inhibitor) vs. normalized response—variable slope) generated in GraphPad Prism 9.0.0 program (Graph Pad, San Diego, CA, USA).

### Degradation assay in human serum

Serum stability studies of compounds **1**–**4** and **11**–**12** were performed in pooled human serum (off the clot) obtained from Innovative Research, Inc (Novi, MI, USA). Stock solutions of the peptidomimetics (10 µl, C = 168 µmol/ml) were diluted into human serum (690 µl) to give an incubation concentration of 2.4 µmol/ml of each peptidomimetic. All samples were incubated at 37 °C and 50 µl aliquots were withdrawn at selected time intervals (for **1**, **3** and **11**: 0 min, 4 h, 8 h, 12 h, 24 h, 30 h, 36 h, 48 h, 60 h, 72 h and 96 h; for **2**, **4** and **12**: 0 min, 12 h, 24 h, 36 h, 48 h, 60 h, 72 h, 84 h, 96 h, 120 h and 144 h). Then 200 µl of ACN/H_2_O/FA (89:9:2, v/v) was added to the samples in order to precipitate the serum proteins. The cloudy mixtures were vortexed, cooled to 4 °C and subsequently centrifuged (14,000 rpm) for 15 min in 4 °C to pellet the proteins. Every time, 150 µl of supernatant was collected, then diluted with water and freeze-dried. The freeze-dried samples were redissolved in 0.1% TFA (or FA for LC–MS analyses), then analysed by RP-HPLC or RP-HPLC/MS (for selected samples), (Supplemental Figures SI-26 to SI-31). As internal standards, H-Trp-OH and Z-Lys-OH were used. The samples were tested in four independent experiments, each performed once (3× for RP-HPLC and one for LC–MS). All experiments were conducted in sterile Eppendorf tubes and sampled with sterile tips, using high-purity organic solvents and Milli-Q quality water. Before the peptidomimetics were tested, the metabolic activity of human serum was checked first using endomorphin-2 (EM-2: Tyr-Pro-Phe-Phe-NH_2_), at the concentration identical to that for peptidomimetics, (Supplemental Figure SI-32). In the case of EM-2, the metabolic activity was tested in three independent experiments.

## Results

### Design rationale

As mentioned, in the presented studies we wanted to obtain more stable and active analogues of our branched peptidomimetic inhibitors **1** and **2** (Table 1). To this end, we considered replacements of the C-terminal arginine with its mimetics, including those in Table 1. Our designs were supported by structure-based considerations and molecular docking.

**Table 1:**
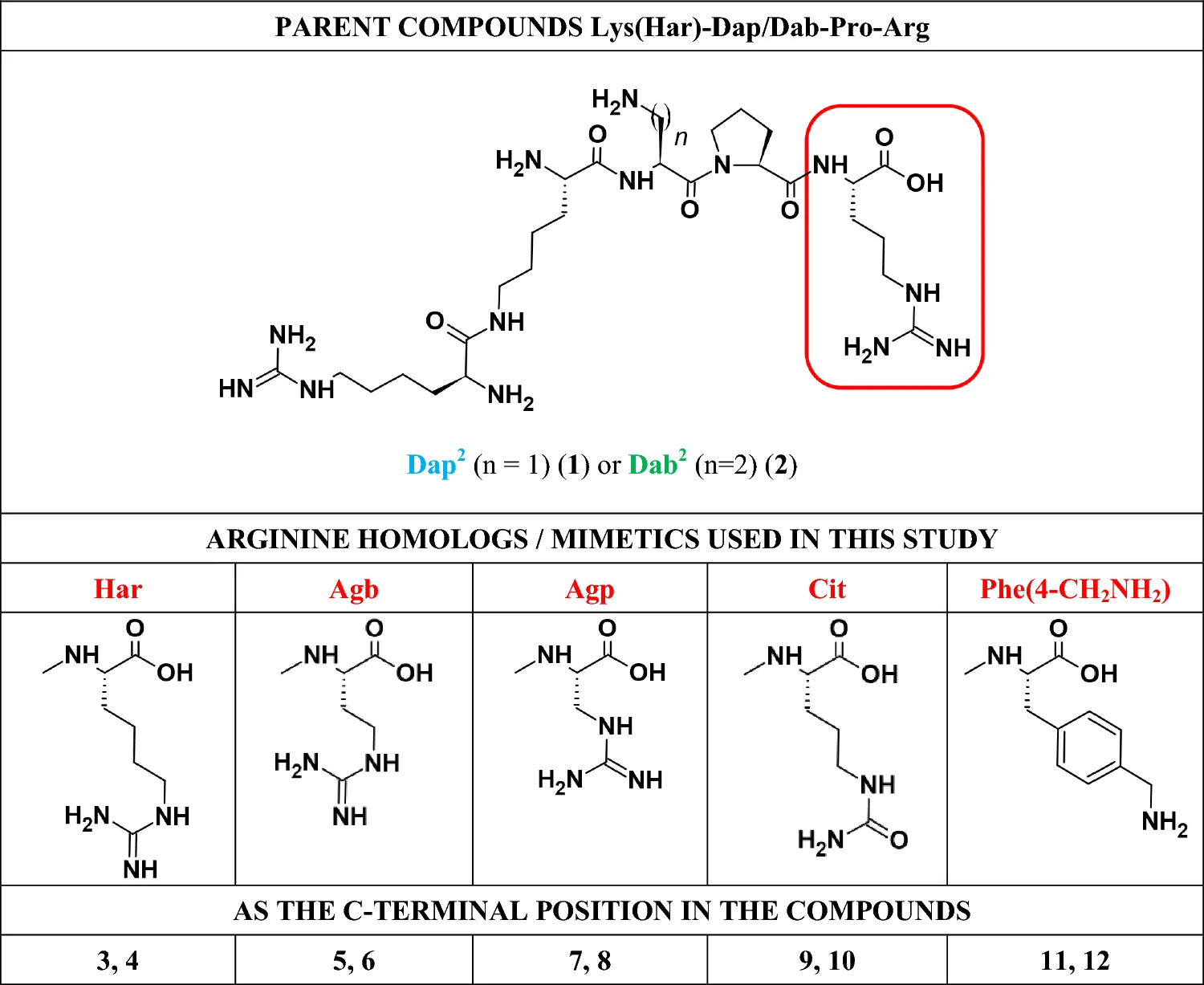
Structure of parent compounds, and arginine homologs/mimetics used in the C-terminal position (in the red square frame)

**Fig. 1:**
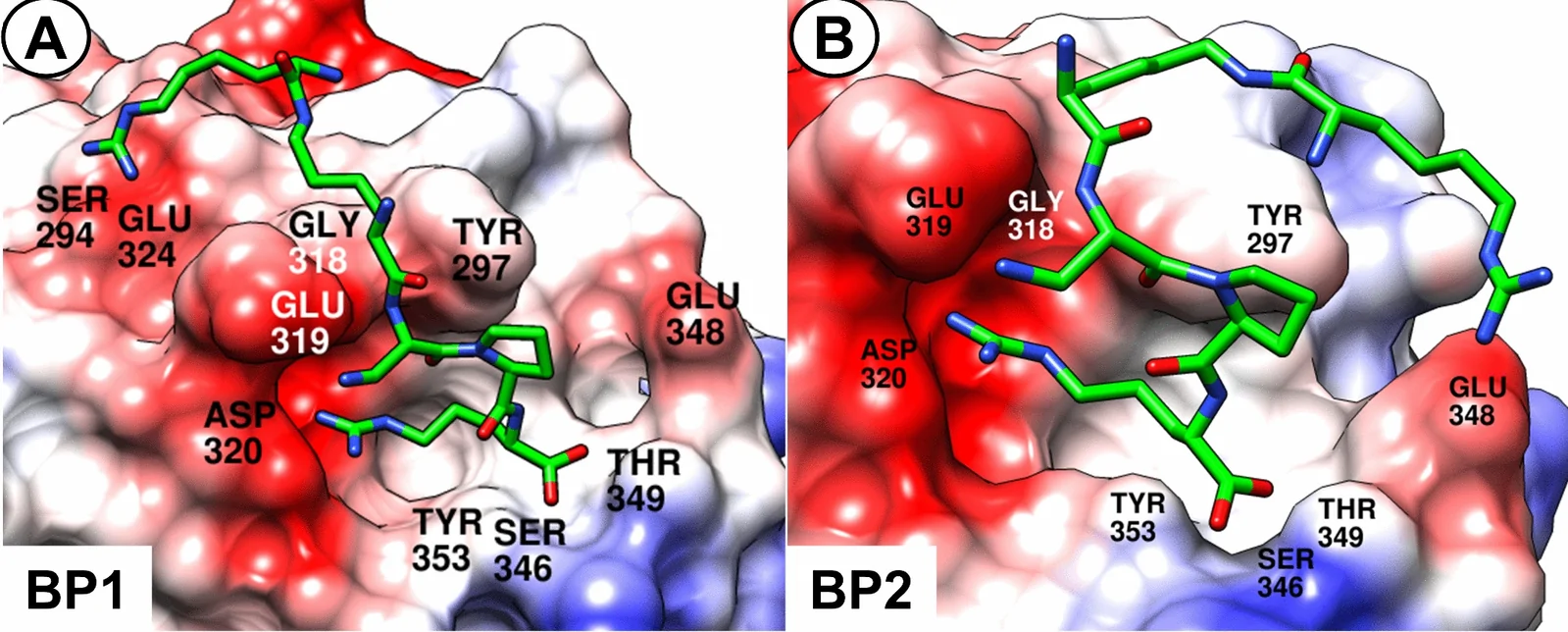
Two dominant binding poses (BP1 and BP2) found in molecular dynamics simulations for **1** in complex with NRP-1. The protein is depicted as an electrostatic color-coded surface (red: negative charges, white: neutral, blue: positive). Colors of the ligand are green, red and blue for carbon, oxygen and nitrogen, respectively

In our previous work (Tymecka et al. 2018), based on molecular dynamics simulations, we proposed an interaction model for compounds **1** and **2** bound to NRP-1. The model had the following key features: (1) the branched peptidomimetics adopt more than one binding pose (BP), with two poses (BP1 and BP2, Fig. 1) being dominant and in mutual equilibrium; (2) the C-terminal Arg residue is inserted in the shallow cleft at the NRP-1 surface and forms several interactions, including H-bonds to Asp320, Ser346, Thr349 (Fig. 1); (3) the middle and N-terminal parts of the peptidomimetic retain some residual mobility and switch between positioning BP1 and BP2; (4) they form permanent or transient H-bonds to several residues out of Gly318, Glu319, Glu324, Ser294, Tyr297, Glu348 (the exact set of interaction partners depends on the binding pose).

In the light of this model, we contemplated extending the Arg^4^ residue to Har. According to crystal structure 5IJR (Mota et al. 2018), Har can be accommodated in the cleft although with some displacement of the binding mode compared to if Arg is present in the cleft. Based on our modelling (Supplemental Figures SI-5 to SI-8), we assumed that interactions of Lys(Har)^1^ and Dap/Dab^2^ would not be much affected, so they should allow retaining significant affinity.

Furthermore, we speculated that it should be also possible to shorten the Arg side chain to 2-amino-4-guanidino-butyric acid (Agb) or even 2-amino-3-guanidino-propionic acid (Agp). Given that Lys(Har)^1^ and Dap/Dab^2^ side-chains are long and flexible, it was envisaged that some shortening in Xaa^4^ should be tolerated. In that case, the first and second residue should still be able to reach their interaction partners. Meanwhile, the shorter Xaa^4^ should be still able to interact with Asp320 and/or Ser346/Thr349 (Supplemental Figures SI-9 to SI-16).

A kind of an acid-test to the importance of guanidine-Asp320 interactions was provided by a Cit^4^-analogues. According to our modelling, Cit^4^ side chain should enable forming one H-bond to Asp320 (but contrary to other analogues, without charge-assistance; Supplemental Figures SI-17 to SI-20). The Cit^4^-analogues were furthermore quite decently scored by AutoDock Vina (Supplemental Table SI-1), so it was expected that significant affinity would be retained.

In another attempt, it was interesting to see if the C-terminal residue with an aromatic ring could gain some affinity due to partial rigidification and formation of aromatic interactions with Tyr297 (Supplemental Figures SI-21 to SI-24). This consideration led to analogues with C-terminal Phe(4-CH_2_-NH_2_).

**Fig. 2:**
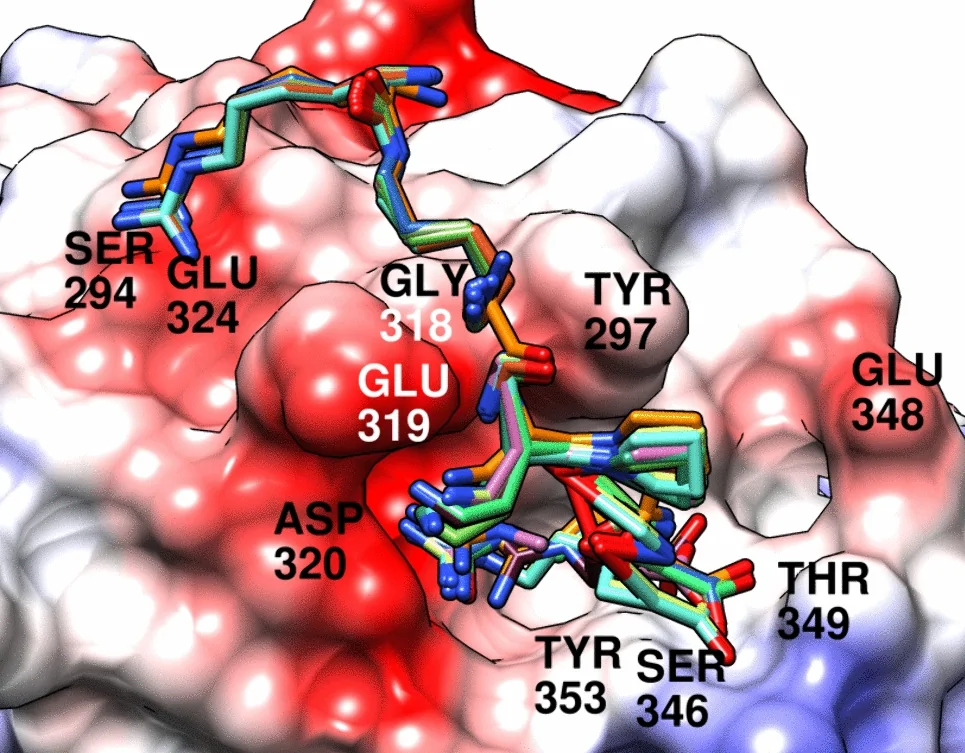
Superposition of compounds **1**–**12** modelled in BP1. The protein is depicted as an electrostatic color-coded surface (red: negative charges, white: neutral, blue: positive). The ligands are shown as coloured sticks

Overall, our conclusion was that the proposed modifications do not disturb the overall binding mode to much extent (Fig. 2), so drastic affinity drops would not occur. At the same time, we believed the unnatural amino-acid in the C-terminal position could improve the metabolic stability.

### Inhibitory activity

In order to verify the accuracy of the design assumptions, a modified competitive enzyme-linked immunosorbent assay (ELISA) was used to evaluate the inhibitory activity of the designed peptidomimetics against VEGF-A_165_/NRP-1 complex formation. In addition to the parent compounds (**1** and **2**), heptapeptide A7R was used for the sake of comparison. The results are summarized in Table 2.

**Table 2:**
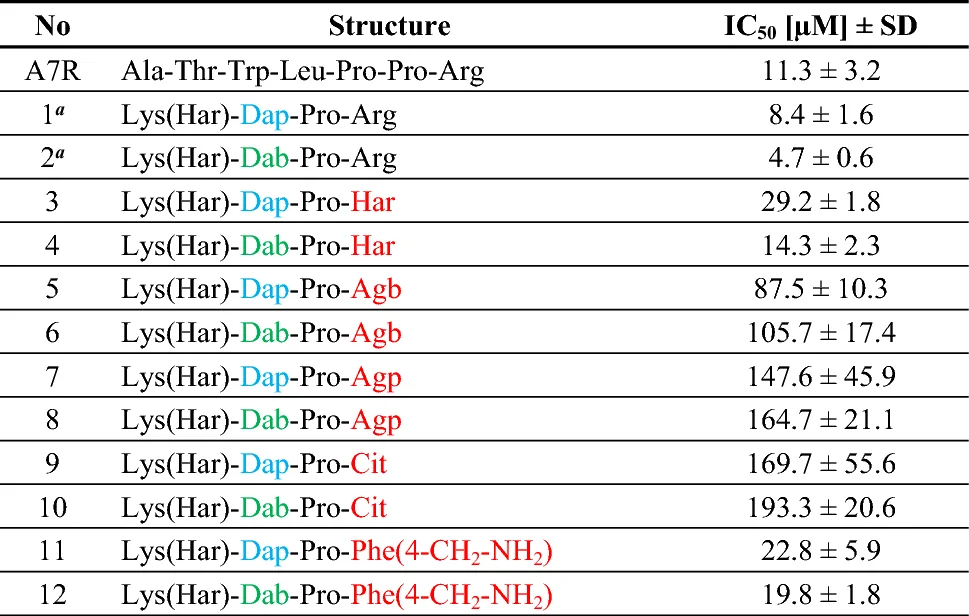
Inhibitory activity of compounds 1–12 and A7R on (bt)-VEGF-A_165_ binding to NRP-1

^a^Parent compounds, Compounds were tested in the concentrations range 100–3.12 μM

Obtained results showed that extension of Arg^4^ side chain (**1** and **2**) by introducing one methylene group to obtain Har^4^ (**3** and **4**, respectively), gives only a slight decrease in inhibitory activity (threefold) as compared to the parent compounds. On the other hand, replacing Arg^4^ with Agb that has side chain shorter by one methylene group leads to compounds **5** and **6** with a 10- and 20-fold weaker inhibition of VEGF-A_165_ binding to NRP-1, respectively (IC_50_ = 87 and 106 μM). Unfortunately, further shortening of Arg^4^ side chain to Agp (− 2 × CH_2_), for **7** and **8**, results in a further significant decrease in inhibitory activity (IC_50_ = 148 and 165 μM, respectively). Moreover, a substantial decrease in the inhibition of VEGF-A_165_/NRP-1 complex formation (close to that found for **7** and **8**) was also observed for compounds **9** and **10** where the guanidine group of Arg was replaced by urea group of Cit (IC_50_ = 170 and 193 μM, respectively). Interestingly, the replacement of the alkyl side chain of Arg^4^ by the aromatic ring of Phe(4-CH_2_-NH_2_)^4^ with a simultaneous change of the guanidine to an amino group (**1** and **2** *vs.* **11** and **12**, respectively), resulted in a slight decrease in activity, comparable to that found for Har^4^-analogues.

### Proteolytic stability in human serum

**Fig. 3:**
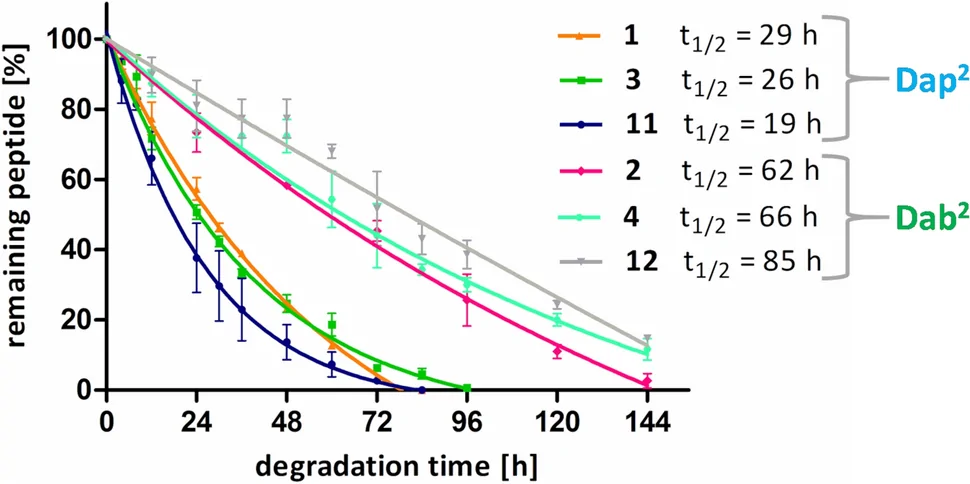
Time-dependent changes in the percentage of the remaining peptidomimetic subjected to proteolytic degradation in human serum

To evaluate the effect of arginine substitution with its mimetics on proteolytic stability, the most potent novel compounds (**3**–**4** and **11**–**12**) and the parent compounds (**1**–**2**) were incubated in human serum at 37 °C. The enzymatic degradation progress was checked for each sample at specific time intervals by RP-HPLC and the changes in the area of peptide peak were determined, where the area at time t_0_ was treated as 100%. The results are depicted in Fig. 3.

In general, all the tested compounds were degraded, but the process occurred at different rates depending on the structure of the compound. The peptidomimetics containing Dap residue at position 2 (compounds **1**, **3** and **11**) were less stable. Their estimated half-life times were t_½_ = 29 h (**1**), t_½_ = 26 h (**3**) and t_½_ = 19 h (**11**). Interestingly, the compounds containing Dab residue at the second position turned out to be more stable with half-life times of 62 or 66 h for **2** (Arg^4^) or **4** (Har^4^), respectively, and even 85 h for **12** (Phe(4-CH_2_-NH_2_)^4^).

**Fig. 4:**
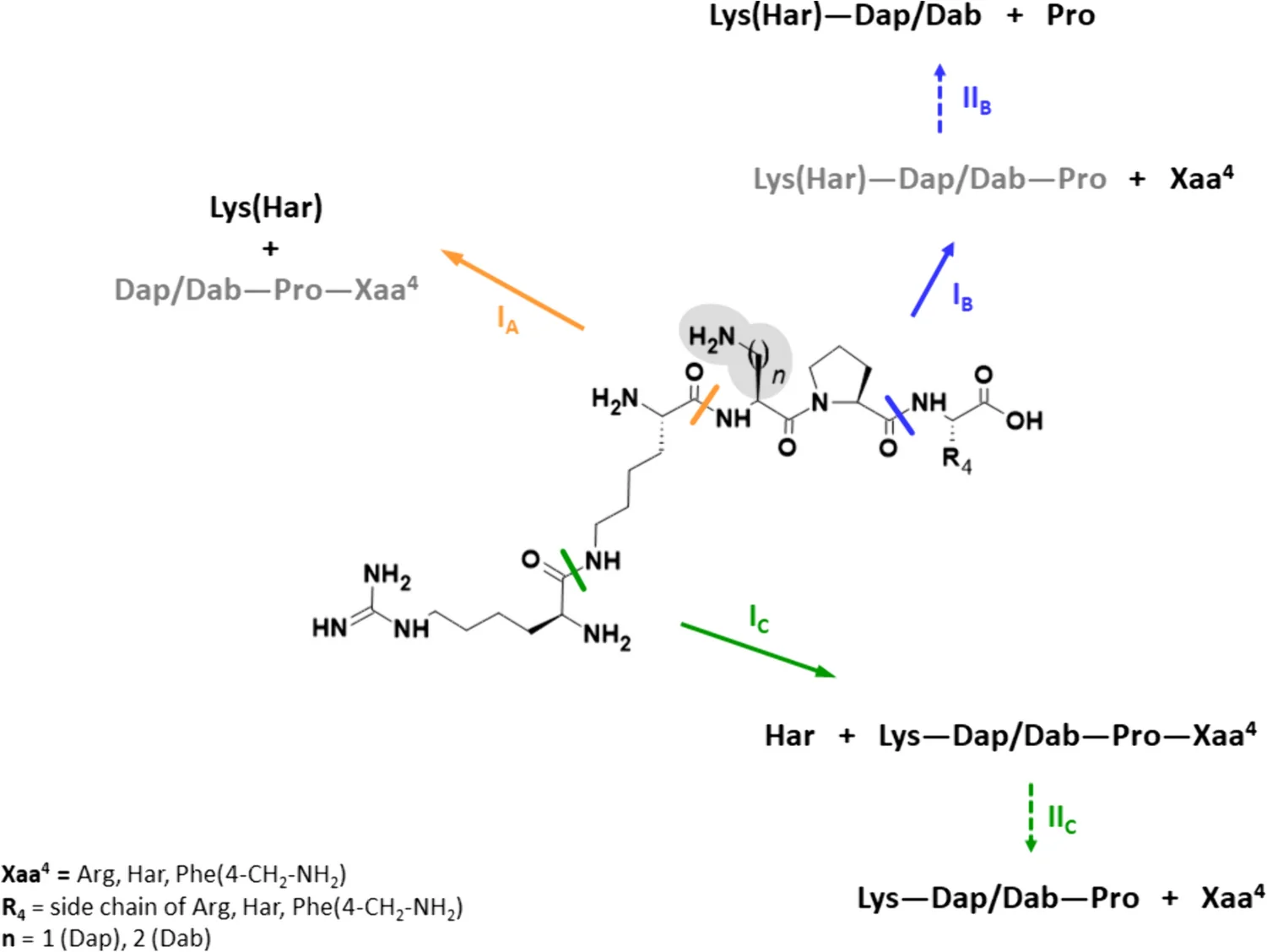
Probable degradation pathways and cleavage sites of **1**–**4** and of **11**–**12**. The general structure of these compounds is shown with marked locations of the first major enzymatic cleavage sites (for pathway *I*_*A*_, *I*_*B*_ and *I*_*C*_). The second position of Dap or Dab (important in view of the half-life time) is highlighted in pale grey. Peptide sequences in grey have not been identified by mass spectrometry. *II*_*B*_ and *II*_*C*_ indicate further degradation pathways for Lys(Har)-Dap/Dab-Pro and Lys-Dap/Dab-Pro-Xaa peptides, respectively

To identify the formed metabolites, and therefore possible degradation pathways, the taken samples were analysed by LC–MS. In case of all compounds, we were able to find similar and/or identical metabolites suggesting three independent and parallel metabolic pathways (Fig. 4). In the first one (I_A_, Fig. 4), as we previously observed (Tymecka et al. 2018), proteolytic enzymes hydrolysed the N-terminal peptide bond between the Lys(Har)^1^ and Dap^2^/Dab^2^ residues. This led to the release of the Lys(Har) and the corresponding Dap/Dab-Pro-Xaa tripeptide should be generated. However, in none of these cases, we were able to identify the expected tripeptides. This is probably because they were rapidly degraded to single amino acids, at a rate faster than the initial study sampling time of 4 or 12 h (for **1**, **3** or **11** and for **2**, **4** or **12**, respectively). Conversely, in the second pathway (I_B_, Fig. 4), the C-terminal peptide bond was cleaved to produce Lys(Har)-Dap/Dab-Pro peptide and the corresponding amino acid Xaa (Arg, Har or Phe(4-CH_2_-NH_2_)). Also for this degradation pathway, we did not observe the expected branched tetrapeptides. However, here we were able to detect the products of their further degradation (II_B_, Fig. 4), i.e. Lys(Har)-Dap/Dab peptides and proline. In the case of the third pathway (I_C_, Fig. 4), we assume that the epsilon-peptide bond between the Lys and Har was cleaved first, since we found Har and Lys-Dap/Dab-Pro-Xaa tetrapeptides as metabolites. Moreover, the resulting tetrapeptides were further degraded to Lys-Dap/Dab-Pro and the corresponding amino acid Xaa (II_C_, Fig. 4).

## Discussion

Three major themes emerge from our results. The first one pertains to the role of the arginine residue in binding to NRP-1. C-terminal Arg is present in endogenous interaction partners for NPR-1, including VEGF-A_165_ or certain semaphorins. Almost all active peptide inhibitors reported so far have the C-terminal Arg. Removing this residue or modifying it has been many times shown to bring about drastic activity losses (refer to Supplemental Table SI-4 for structures and activity data relevant for the discussion below). This was the case for such A7R-analogues as des-Arg^7^-A7R, [Lys^7^]-A7R, [Ala^7^]-A7R or A7R-Ala^8^ (Starzec et al. 2007). The tetrapeptides Lys-Pro-Ala-**D-Arg** and Lys-Pro-Ala-**Lys** are significantly less potent than the Lys-Pro-Ala-**Arg** parent inhibitor (Jarvis et al. 2010). Furthermore, even some simple *N*^α^-substituted arginines (e.g. carbamates like *N*^α^-Boc-Arg-OH or *N*^α^-Cbz-Arg-OH) exhibit measurable binding to NRP-1 (Mota et al. 2018). Arg fragment is also present in peptidomimetics designed based directly on the peptide ligands, including the EG00229 (Jarvis et al. 2010) and EG01377 (Powell et al. 2018). On the contrary, a number of small molecular inhibitors, discovered by virtual screening approaches, lack an Arg substructure (or guanidine moiety), yet still exhibit significant inhibitory potencies (Borriello et al. 2014; Starzec et al. 2014; Liu et al. 2018; Peng et al. 2020; Perez-Miller et al. 2021).

With this background, our desire was checking if Arg^4^ in the structures of our leads **1** and **2** could be replaced with Arg analogues (homologs and mimetics) and still allow retaining reasonable levels of inhibitory activity. The modelled binding poses of our designs suggested that these modifications should enable keeping majority of the interactions of the N-terminal and middle part of the structure, so our reasoning was that inhibitory activity drops should be minor, if any. The experimental data turned out more diversified than this expectation. The Arg^4^-parent compounds were not surpassed in inhibitory activity by any of the novel analogues. The shorter Agb^4^ or Agp^4^ residues gave large decreases in activity and so did the Cit^4^ residue lacking the charged side chain. The elongated Har^4^ residue and the aromatic Phe(4-CH_2_-NH_2_)^4^ residue turned out to be rather tolerated, with the activity decreases being moderate. The presented modifications have not been probed in the peptide inhibitors reported so far. In the peptidomimetics area (Supplemental Table SI-4), analogues close to EG00229 having (*S*)-Phe(4-NH-CH(NH_2_) = NH) or (*S*)-Har pieces instead of (*S*)-Arg fragment exhibited major activity reduction (Jarvis et al. 2010), which is in some way (though of course this is not exact comparison) different than what is seen in our peptide inhibitors.

Overall, our experimental data together with the modelled binding poses once again corroborate the importance of the interactions that take place within the Arg binding cleft on the NRP-1 surface. It seems that in the case of peptide inhibitors these intermolecular contacts are “more important” than others that may take place beyond this cleft.

In the second place, let us note that our modelling approach for the designs had several limitations. First, we utilized only the static binding poses and omitted the search element of the docking, assuming that the close analogues should adapt poses that we saw previously in molecular dynamics (Tymecka et al. 2018). This simplification was dictated by the time requirements needed to run MD simulations. On the other hand, it needs to be admitted that it could lead to incorrect description of the interactions because of the limited interaction sampling or inability to capture dynamic processes (like switching the rotameric states of the binding site), and both could affect the energetics estimations. Another limitation of the modelling approach taken is that we did not consider water molecules that are present in the binding site. As a result, our binding poses cannot capture water-mediated interactions. Furthermore, any solvation effects that could be found in MD are also lost. Relevantly to the current problem, Mota et al. showed that the water molecules play important role in the interactions with NRP-1 binding site (Mota et al. 2018). These limitations might significantly affect the docking outcomes and result in the lack of quantitative agreement between the experimental activity and the scoring results. It seems that further work on VEGF-A_165_/NRP-1 inhibitors could benefit from developing a design approach middle-way between full MD and static, local docking.

The third discussion topic pertains to the observed trends in the stability of our inhibitors. Our anticipation was that upon replacing the C-terminal arginine with its mimetics, we should observe significant changes in the half-life times of novel inhibitors. Surprisingly, a much more pronounced influence on the proteolytic stability was associated with the presence of either Dab or Dap in the second position of the sequence. The Dab^2^-analogues had t_1/2_ at least two times greater (t_1/2_ > 60 h) than their Dap^2^-counterparts (t_1/2_ around 20-30 h). On the other hand, Arg^4^/Har^4^ pairs display half-life differences of only a few hours (3–4 h). That may indicate that the introduction of an additional methylene group into the side chain at the C-terminal position (Arg vs. Har) has a negligible effect on the interaction of particular inhibitor with the active site of enzymes responsible for the hydrolysis of our compounds. Unexpectedly, in the Arg^4^/Phe(4-CH_2_-NH_2_)^4^ pairs, the stability change is also not regular however, over a much wider range, being a 10-h-loss in the case of Dap^2^-analogues (**1** vs **11**) while a more than a 20-h-gain in the case of Dab^2^-analogues (**2** vs **12**). This variability causes that stability of compounds in the Dap^2^ series changes as follows Arg^4^ > Har^4^ > Phe(4-CH_2_-NH_2_)^4^ while in the case of the Dab^2^ series it changes in the opposite direction, i.e. Phe(4-CH_2_-NH_2_)^4^ > Har^4^ > Arg^4^. This reversed order of stability surprised us, and we do not know exactly what causes it. A putative explanation could be that the active site of a certain enzyme (or enzymes), that plays the key role in the degradation pathways seen with our inhibitors, adopts its substrates in separate binding modes depending on what is present in position two. These binding modes are associated with the placement of C-terminal residue in two separate sub-pockets of the site. As a result, the stability trends in the two series are notably different. In view of the above results, further systematic studies on the structure-stability relationships of our lead structures are warranted, including an in-depth exploration of the influence of the second position.

## Conclusions

In conclusion, ten novel analogues of Lys(Har)-Dab/Dap-Pro-Arg were designed, synthesized and tested. The scope of molecular modification included variation of the C-terminal residue with Arg mimetics/derivatives. None of the novel analogues was more potent than the parent compounds, but Har^4^ and Phe(4-CH_2_-NH_2_)^4^ residues turned out to be tolerated, yielding only moderate decreases in affinity. Strikingly, in the tested analogues it was position two rather than four that influenced the proteolytic stability more. The Dab^2^-analogues exhibited half-life times beyond 60 h. Our study builds up further SAR knowledge likely to be useful in future research. In our case, when more stable and active inhibitors are developed, we then plan to use them as targeting vector (molecule) in radioconjugates, that can be used as theranostic-like radiopharmaceuticals for the imaging and therapy of cancers that overexpress NRP-1. So far, using Lys(Har)-Dab-Pro-Arg for this purpose turned out suboptimal because of unsatisfactory nano-scale stability in human serum, especially for use as therapeutic radioagents (Masłowska et. al. 2022, 2023). Defining the border pharmacophoric requirements may be helpful in works on both ‘classic’ single-function VEGF-A_165_/NRP-1 inhibitors but also on the recently sought-after multifunctional/multitarget compounds that utilize the NRP-1 binding component, like dual inhibitors of NRP-1 and receptor-binding domain of Spike protein of SARS-CoV-2 (Hu et al. 2023) or dual inhibitors of NRP-1 and tubulin (Zheng et al. 2023).

## Supplementary Information

Below is the link to the electronic supplementary material.

Supplementary file1 (PDF 3081 KB)

## Data Availability

No datasets were generated or analysed during the current study.
