# Supplementary material for: Peptidomimetic inhibitors of the VEGF-A165/NRP-1 complex obtained by modification of the C-terminal arginine
Source: Amino Acids. 2024 Aug 24;56(1):49. doi: 10.1007/s00726-024-03411-8 (PMC11344719; doi:10.1007/s00726-024-03411-8)
Supplement: Supplementary file 1 — Supplementary file1 (PDF 3081 KB) [file 726_2024_3411_MOESM1_ESM.pdf]

Supplementary Information

to:

Peptidomimetic inhibitors of the VEGF-A$_{165}$ / NRP-1 complex obtained by modification of the C-terminal arginine

Dagmara Tymecka [1], Patrycja Redkiewicz [2], Piotr F.J. Lipiński [2] and Aleksandra Misicka [1]

[1] Faculty of Chemistry, University of Warsaw, Pasteura 1, 02-093 Warsaw, Poland;

[2] Department of Neuropeptides, Mossakowski Medical Research Institute Polish Academy of Sciences, Pawińskiego 5, 02–106 Warsaw, Poland;

ORCID ID: Dagmara Tymecka: 0000-0002-8909-4087, Patrycja Redkiewicz: 0000-0001-8654-580X, Piotr F.J. Lipiński: 0000-0002-8364-7955, Aleksandra Misicka: 0000-0001-7446-2396

***** Correspondence: dulok@chem.uw.edu.pl (D.T.); misicka@chem.uw.edu.pl (A.M.)

Table of contents

Table SI-1 AutoDock Vina scoring for compounds **1–12**.
Figure SI-1. Compound 1 modelled in Binding Pose 1 (BP1).
Figure SI-2. Compound 1 modelled in Binding Pose 2 (BP2).
Figure SI-3. Compound 2 modelled in Binding Pose 1 (BP1).
Figure SI-4. Compound 2 modelled in Binding Pose 2 (BP2).
Figure SI-5. Compound 3 modelled in Binding Pose 1 (BP1).
Figure SI-6. Compound 3 modelled in Binding Pose 2 (BP2).
Figure SI-7. Compound 4 modelled in Binding Pose 1 (BP1).
Figure SI-8. Compound 4 modelled in Binding Pose 2 (BP2).
Figure SI-9. Compound 5 modelled in Binding Pose 1 (BP1).
Figure SI-10. Compound 5 modelled in Binding Pose 2 (BP2).
Figure SI-11. Compound 6 modelled in Binding Pose 1 (BP1).
Figure SI-12. Compound 6 modelled in Binding Pose 2 (BP2).
Figure SI-13. Compound 7 modelled in Binding Pose 1 (BP1).
Figure SI-14. Compound 7 modelled in Binding Pose 2 (BP2).
Figure SI-15. Compound 8 modelled in Binding Pose 1 (BP1).
Figure SI-16. Compound 8 modelled in Binding Pose 2 (BP2).
Figure SI-17. Compound 9 modelled in Binding Pose 1 (BP1).
Figure SI-18. Compound 9 modelled in Binding Pose 2 (BP2).
Figure SI-19. Compound 10 modelled in Binding Pose 1 (BP1).
Figure SI-20. Compound 10 modelled in Binding Pose 2 (BP2).
Figure SI-21. Compound 11 modelled in Binding Pose 1 (BP1).
Figure SI-22. Compound 11 modelled in Binding Pose 2 (BP2).
Figure SI-23. Compound 12 modelled in Binding Pose 1 (BP1).
Figure SI-24. Compound 12 modelled in Binding Pose 2 (BP2).

Table SI-2: Physical Characteristics of peptidomimetics – **part 1**

High resolution mass spectra were acquired on the Shimadzu LCMS-9030 mass spectrometer with electrospray ionization (ESI). Phase A: water + 0.03 % of FA, phase B: methanol and linear elution 50 % of phase B was used with flow 0.4 ml/min.

Table SI-3: Physical Characteristics of peptidomimetics – **part 2**

Analytical RP-HPLC traces were acquired on the Shimadzu prominence LC-20 system using Jupiter Proteo C12 column (90 A, 250 x 4.6 mm, 4 µm; Phenomenex). Phase A: water + 0.1 % of TFA, phase B: acetonitrile + 0.1 % of TFA; non-linear gradient was applied: 0–15% B for 20 min followed by an increase to 70% B from 20 to 26 min with flow 1 ml/min.

Figure SI-25: Analytical RP-HPLC traces (absorbance at 215 nm) of all peptidomimetics.

Analytical RP-HPLC traces were acquired on the Shimadzu prominence LC-20 system using Jupiter Proteo C12 column (90 A, 250 x 4.6 mm, 4 µm; Phenomenex). Phase A: water + 0.1 % of TFA, phase B: acetonitrile + 0.1 % of TFA; non-linear gradient was applied: 0–15% B for 20 min followed by an increase to 70% B from 20 to 26 min with flow 1 ml/min.

Figures SI-26 to SI-31: RP-HPLC traces and HR-MS results recorded for selected time points during stability tests of Lys(Har)-Dap/Dab-Pro-Xaa (**1-4 and 11-12**).

Analytical RP-HPLC traces were acquired on the Shimadzu prominence LC-20 system using Jupiter Proteo C12 column (90 A, 250 x 4.6 mm, 4 µm; Phenomenex). Phase A: water + 0.1 % of TFA, phase B: acetonitrile + 0.1 % of TFA; non-linear gradient was applied: 0–20% B for 20 min followed by an increase to 95% B from 20 to 38 min with flow 1 ml/min.

Mass spectra of the degradation products were acquired on the Shimadzu LCMS-9030 mass spectrometer with electrospray ionization (ESI), where the Arion Polar C18 column (250 x 4.6 mm, 5 µm; Chromservis s.r.o.) was used. Phase A: solution consisting of 15 mM ammonium formate and 0.1% formic acid in water, phase B: acetonitrile; non-linear gradient was applied: 0–12% B for 16 min followed by an increase to 95% B from 16 to 36 min with flow 0.4 ml/min.

Figure SI-32: RP-HPLC traces recorded for selected time points during serum activity tests with Tyr-Pro-Phe-Phe-NH_2 (EM-2)
Analytical RP-HPLC traces were acquired on the Shimadzu prominence LC-20 system using Jupiter Proteo C12 column (90 A, 250 x 4.6 mm, 4 µm; Phenomenex). Phase A: water + 0.1 % of TFA, phase B: acetonitrile + 0.1 % of TFA; non-linear gradient was applied: 0–20% B for 20 min followed by an increase to 95% B from 20 to 38 min with flow 1 ml/min.

Table SI-4. Selected inhibitors of the VEGF-A_{165}/NRP-1 interaction (or NRP-1 ligands), illustrative for the role of C-terminal Arg residue/fragment.

References (only for supplementary information)

Table SI-1. AutoDock Vina scoring for compounds **1–12**.

**Cmpd**	**Residues in position**		**Nrot**	**Vina scoring [kcal/mol]**			
				**BP1**[1]		**BP2**[2]	
	2	4		Default scoring function	Without the N_{rot} penalty[3]	Default scoring function	Without the N_{rot} penalty
**1**	Dap	Arg	25	-3.7	-8.8	-2.0	-4.8
**2**	Dab	Arg	26	-2.8	-6.8	-1.6	-4.0
**3**	Dap	Har	26	-1.9	-4.5	-2.1	-4.9
**4**	Dab	Har	27	-1.6	-4.0	-1.7	-4.3
**5**	Dap	Agb	24	-2.3	-5.3	-2.6	-5.9
**6**	Dab	Agb	25	-2.4	-5.6	-2.3	-5.6
**7**	Dap	Agp	22	-1.6	-3.6	-1.5	-3.4
**8**	Dab	Agp	23	-1.6	-3.7	-1.2	-2.9
**9**	Dap	Cit	25	-2.9	-6.9	-1.8	-4.5
**10**	Dab	Cit	26	-2.9	-7.1	-1.5	-3.6
**11**	Dap	Phe(4-CH_2NH_2)	25	-1.7	-4.0	-2.2	-5.2
**12**	Dab	Phe(4-CH_2NH_2)	26	-2.3	-5.5	-1.5	-3.7

[1]BP1 – binding pose 1, [2]BP2 – binding pose 2, [3]N_{rot} – number of rotatable bonds, for the equation of the scoring function refer to: (Trott and Olson 2010)

Figure SI-1. Compound 1 modelled in Binding Pose 1 (BP1).

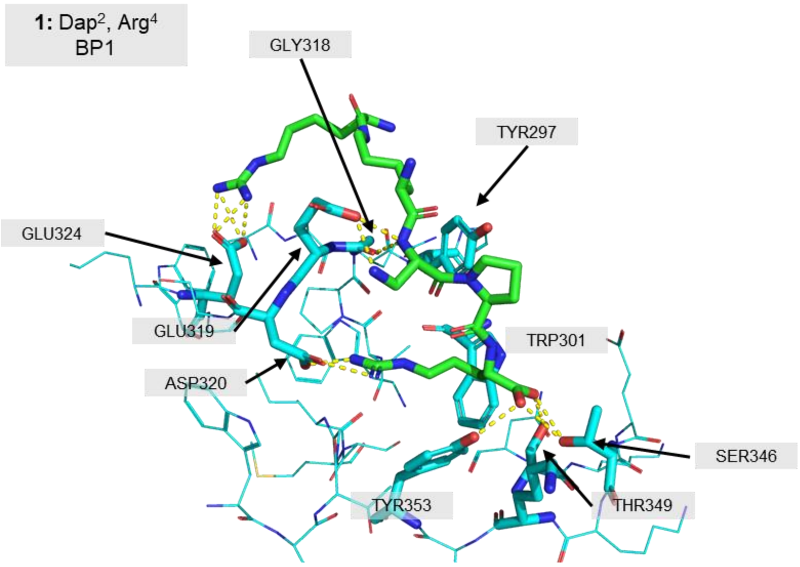

Protein in blue, ligand in green. Hydrogen display suppressed. Only selected residues of the protein are shown or labelled. Yellow dashed lines mark donor-acceptor pairs at the H-bonding distance (<3.2 Å)

Figure SI-2. Compound 1 modelled in Binding Pose 2 (BP2).

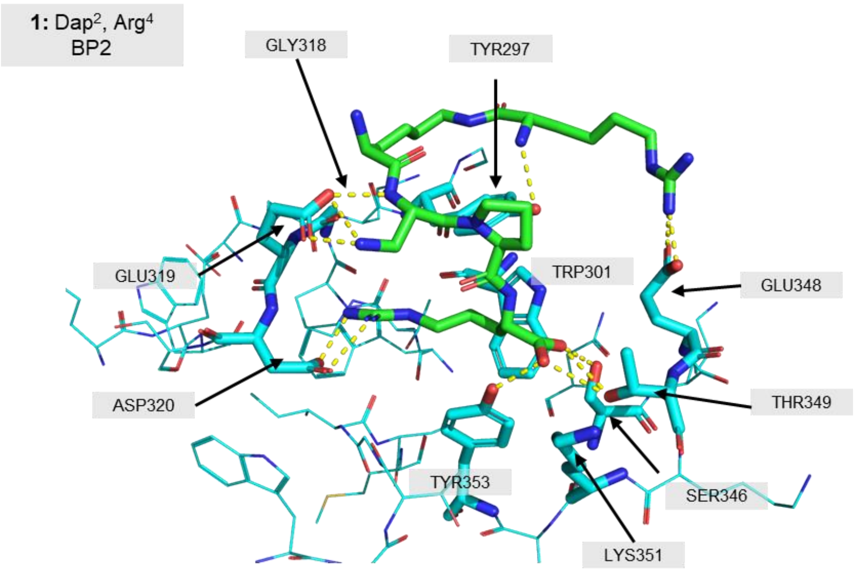

Protein in blue, ligand in green. Hydrogen display suppressed. Only selected residues of the protein are shown or labelled. Yellow dashed lines mark donor-acceptor pairs at the H-bonding distance (<3.2 Å)

Figure SI-3. Compound 2 modelled in Binding Pose 1 (BP1).

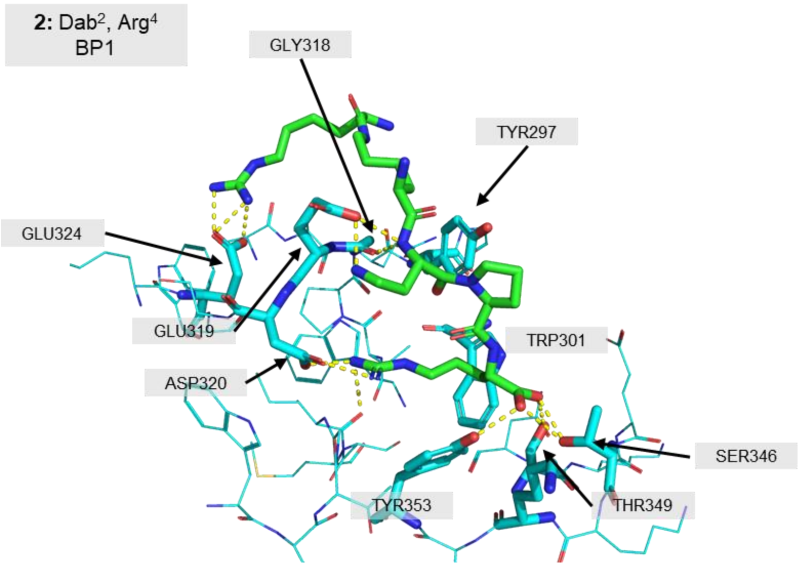

Protein in blue, ligand in green. Hydrogen display suppressed. Only selected residues of the protein are shown or labelled. Yellow dashed lines mark donor-acceptor pairs at the H-bonding distance (<3.2 Å)

Figure SI-4. Compound 2 modelled in Binding Pose 2 (BP2).

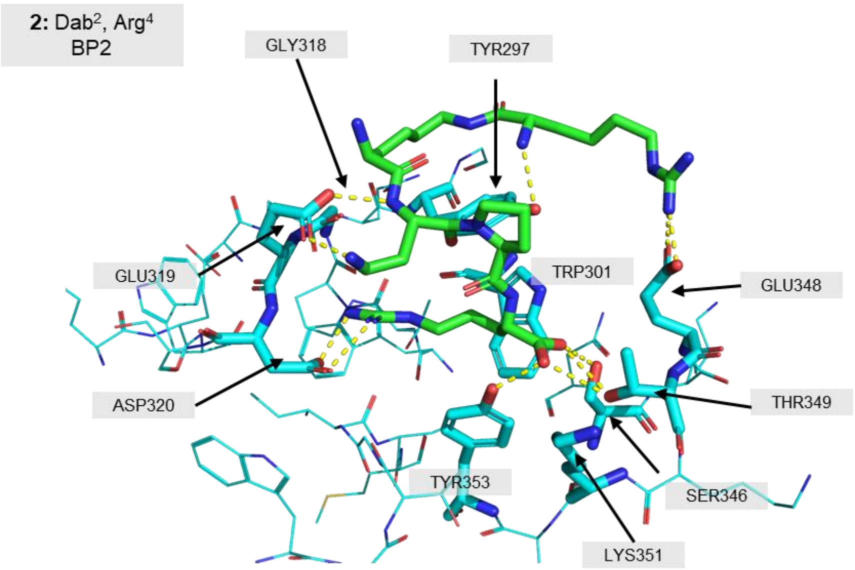

Protein in blue, ligand in green. Hydrogen display suppressed. Only selected residues of the protein are shown or labelled. Yellow dashed lines mark donor-acceptor pairs at the H-bonding distance (<3.2 Å)

Figure SI-5. Compound 3 modelled in Binding Pose 1 (BP1).

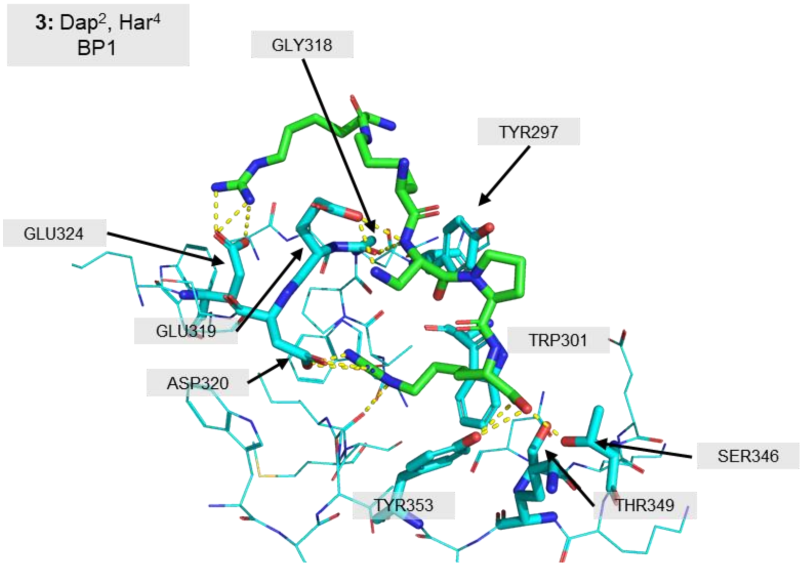

Protein in blue, ligand in green. Hydrogen display suppressed. Only selected residues of the protein are shown or labelled. Yellow dashed lines mark donor-acceptor pairs at the H-bonding distance (<3.2 Å)

Figure SI-6. Compound 3 modelled in Binding Pose 2 (BP2).

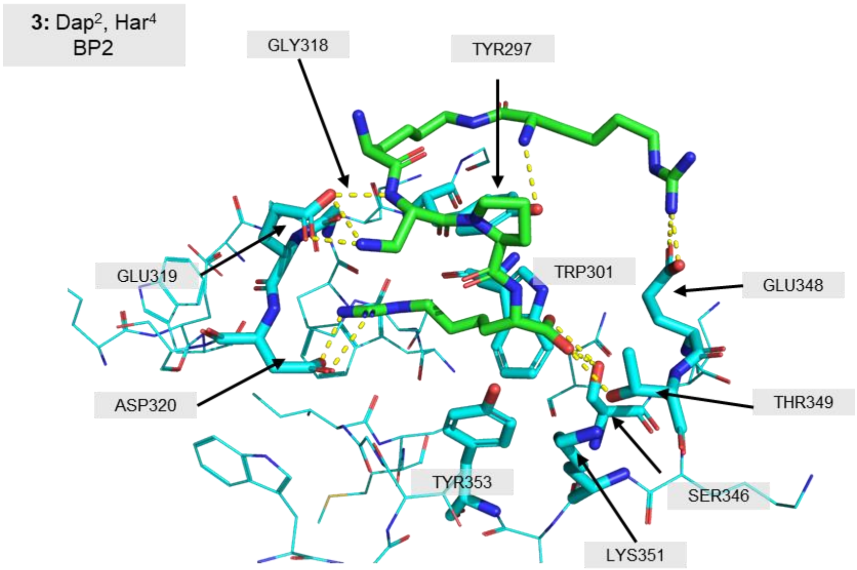

Protein in blue, ligand in green. Hydrogen display suppressed. Only selected residues of the protein are shown or labelled. Yellow dashed lines mark donor-acceptor pairs at the H-bonding distance (<3.2 Å)

Figure SI-7. Compound 4 modelled in Binding Pose 1 (BP1).

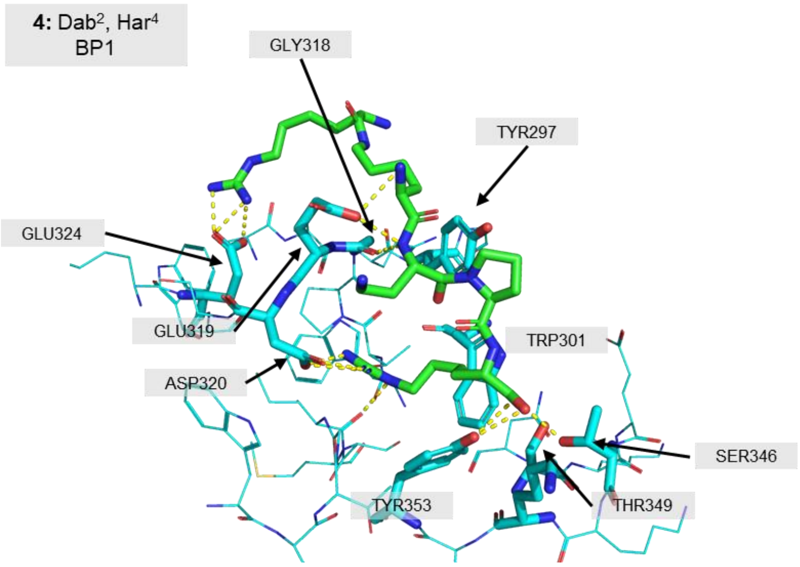

Protein in blue, ligand in green. Hydrogen display suppressed. Only selected residues of the protein are shown or labelled. Yellow dashed lines mark donor-acceptor pairs at the H-bonding distance (<3.2 Å)

Figure SI-8. Compound 4 modelled in Binding Pose 2 (BP2).

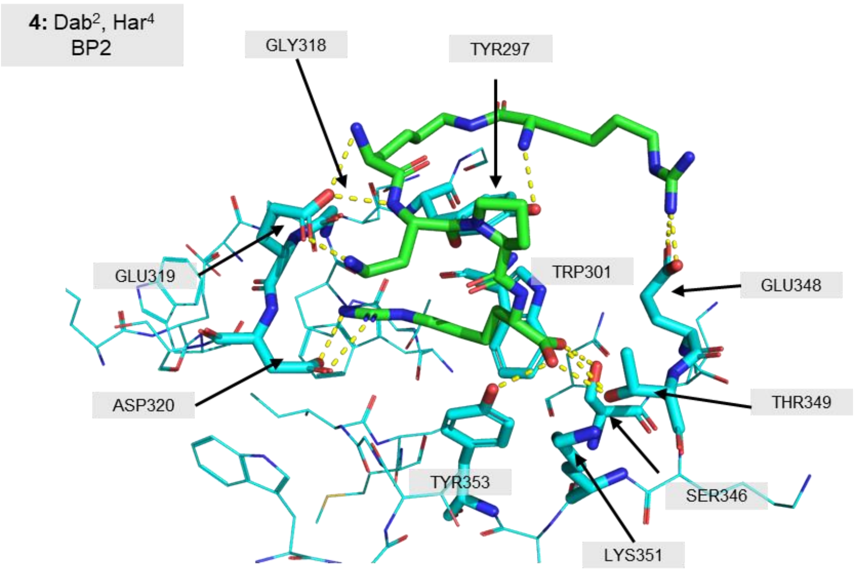

Protein in blue, ligand in green. Hydrogen display suppressed. Only selected residues of the protein are shown or labelled. Yellow dashed lines mark donor-acceptor pairs at the H-bonding distance (<3.2 Å)

Figure SI-9. Compound 5 modelled in Binding Pose 1 (BP1).

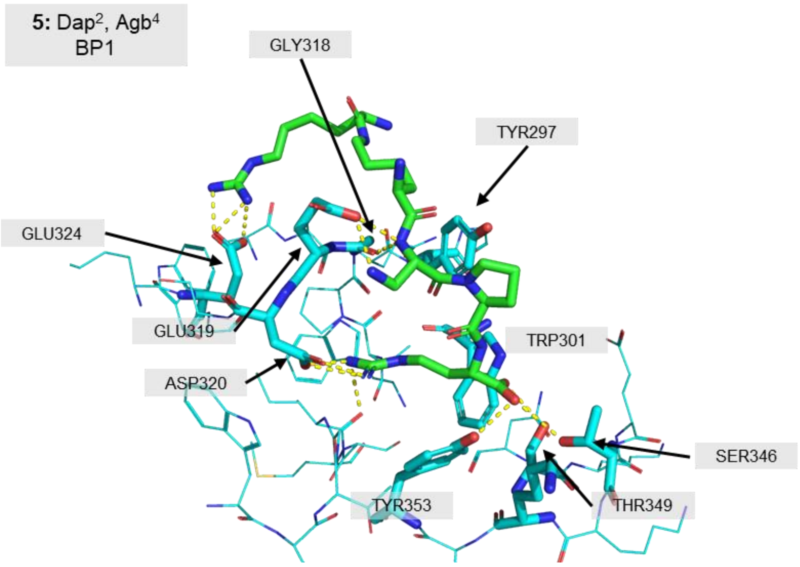

Protein in blue, ligand in green. Hydrogen display suppressed. Only selected residues of the protein are shown or labelled. Yellow dashed lines mark donor-acceptor pairs at the H-bonding distance (<3.2 Å)

Figure SI-10. Compound 5 modelled in Binding Pose 2 (BP2).

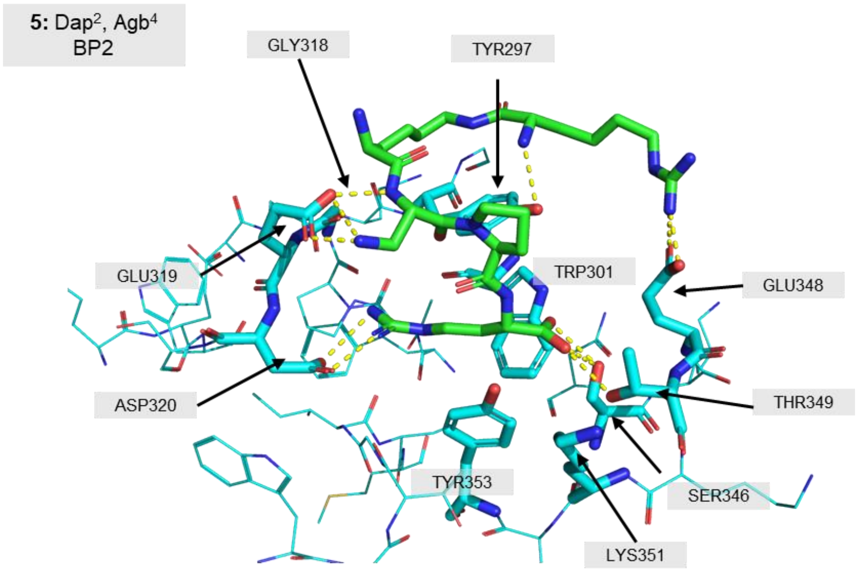

Protein in blue, ligand in green. Hydrogen display suppressed. Only selected residues of the protein are shown or labelled. Yellow dashed lines mark donor-acceptor pairs at the H-bonding distance (<3.2 Å)

Figure SI-11. Compound 6 modelled in Binding Pose 1 (BP1).

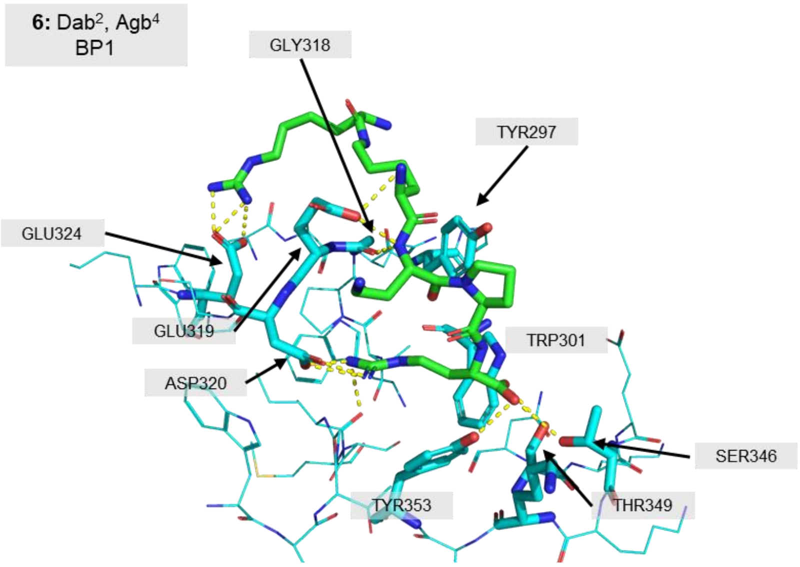

Protein in blue, ligand in green. Hydrogen display suppressed. Only selected residues of the protein are shown or labelled. Yellow dashed lines mark donor-acceptor pairs at the H-bonding distance (<3.2 Å)

Figure SI-12. Compound 6 modelled in Binding Pose 2 (BP2).

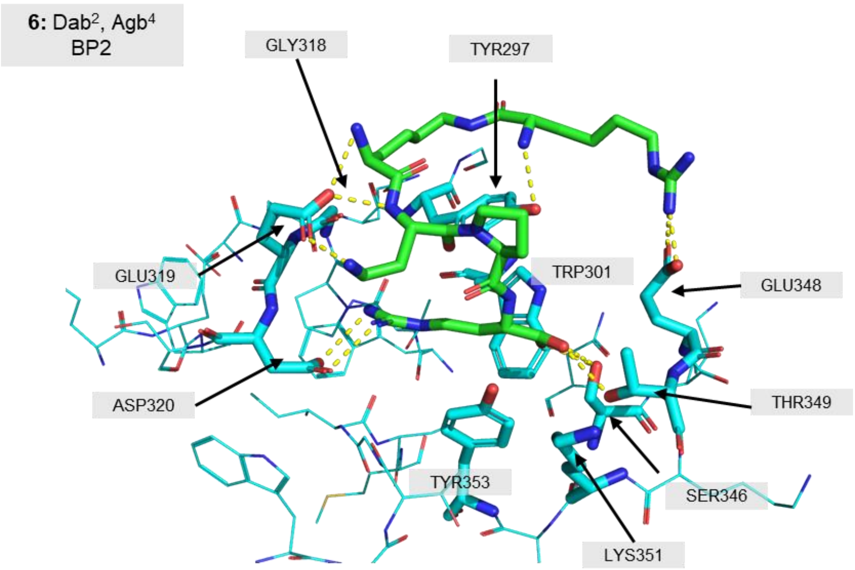

Protein in blue, ligand in green. Hydrogen display suppressed. Only selected residues of the protein are shown or labelled. Yellow dashed lines mark donor-acceptor pairs at the H-bonding distance (<3.2 Å)

Figure SI-13. Compound 7 modelled in Binding Pose 1 (BP1).

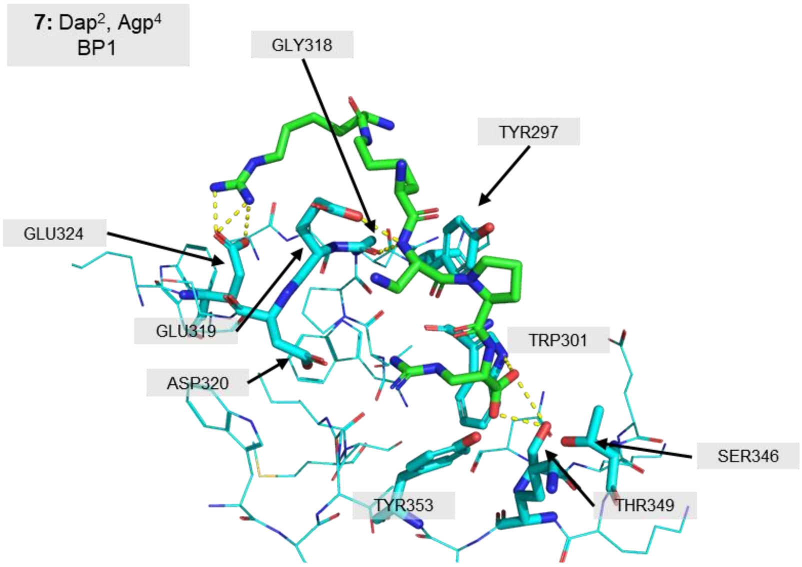

Protein in blue, ligand in green. Hydrogen display suppressed. Only selected residues of the protein are shown or labelled. Yellow dashed lines mark donor-acceptor pairs at the H-bonding distance (<3.2 Å)

Figure SI-14. Compound 7 modelled in Binding Pose 2 (BP2).

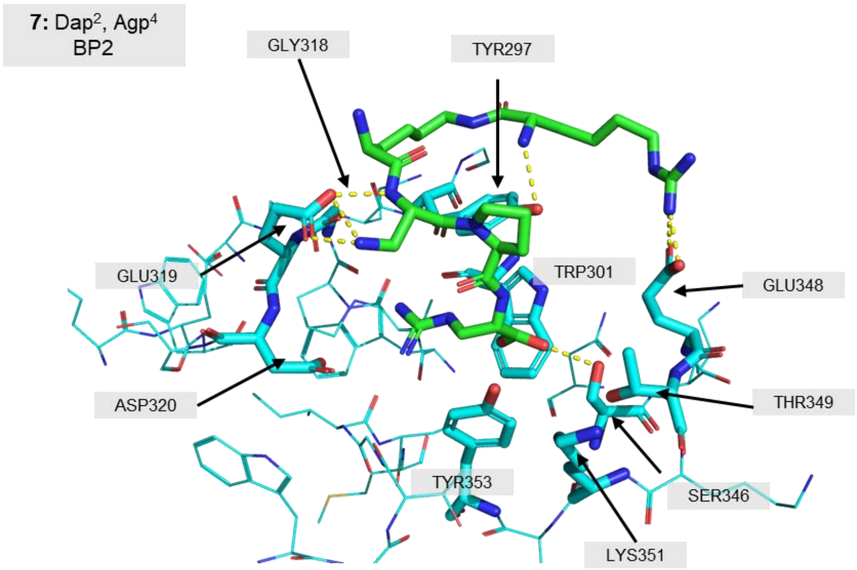

Protein in blue, ligand in green. Hydrogen display suppressed. Only selected residues of the protein are shown or labelled. Yellow dashed lines mark donor-acceptor pairs at the H-bonding distance (<3.2 Å)

Figure SI-15. Compound 8 modelled in Binding Pose 1 (BP1).

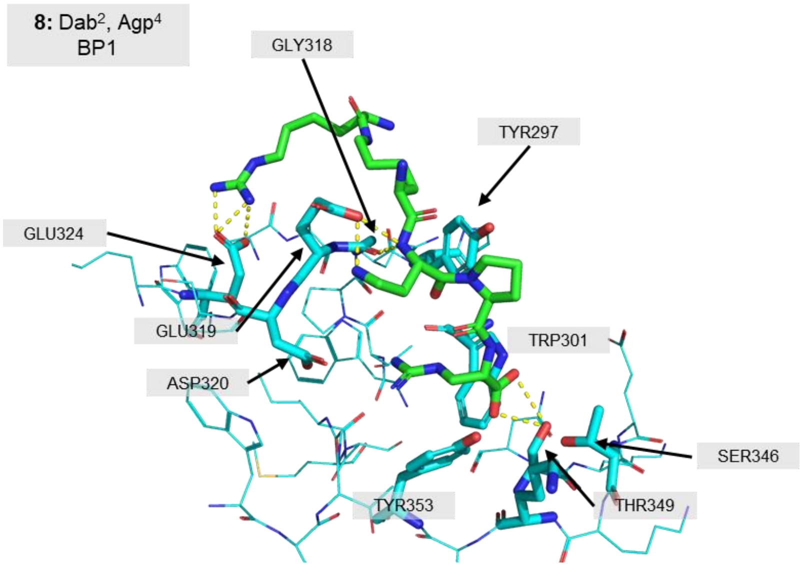

Protein in blue, ligand in green. Hydrogen display suppressed. Only selected residues of the protein are shown or labelled. Yellow dashed lines mark donor-acceptor pairs at the H-bonding distance (<3.2 Å)

Figure SI-16. Compound 8 modelled in Binding Pose 2 (BP2).

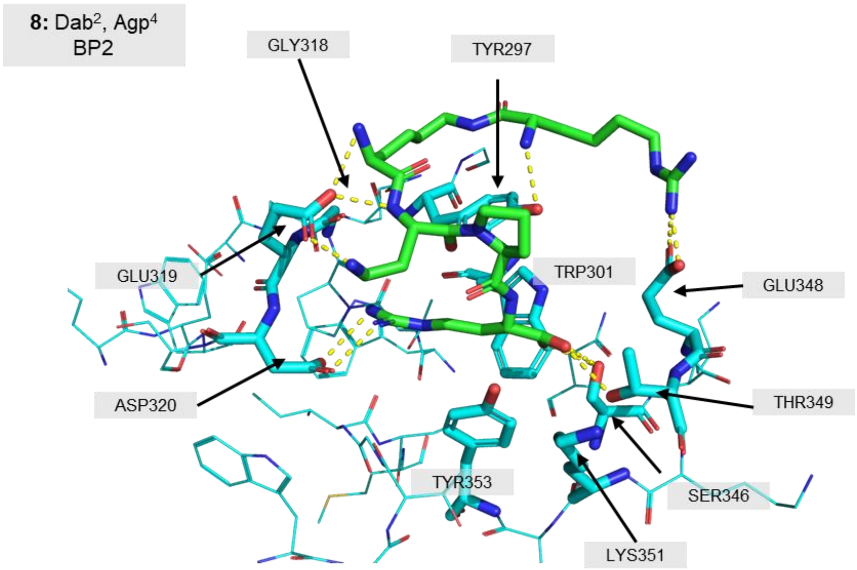

Protein in blue, ligand in green. Hydrogen display suppressed. Only selected residues of the protein are shown or labelled. Yellow dashed lines mark donor-acceptor pairs at the H-bonding distance (<3.2 Å)

Figure SI-17. Compound 9 modelled in Binding Pose 1 (BP1).

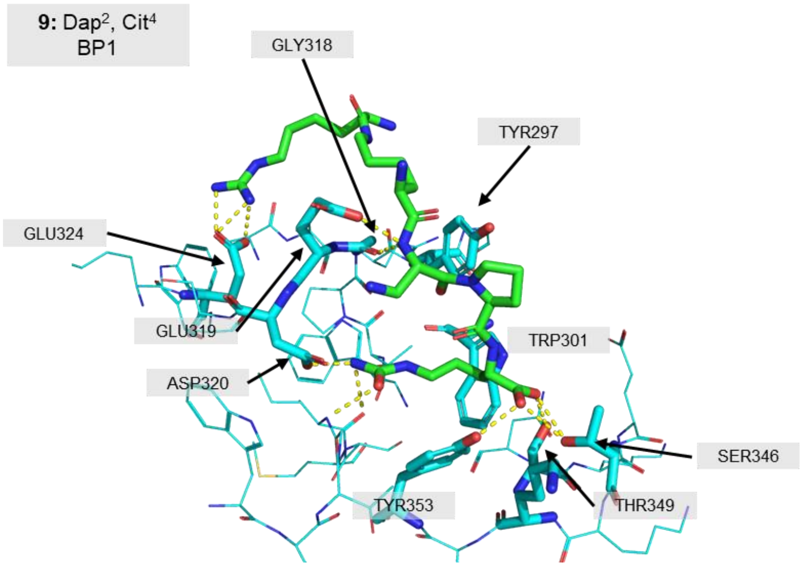

Protein in blue, ligand in green. Hydrogen display suppressed. Only selected residues of the protein are shown or labelled. Yellow dashed lines mark donor-acceptor pairs at the H-bonding distance (<3.2 Å)

Figure SI-18. Compound 9 modelled in Binding Pose 2 (BP2).

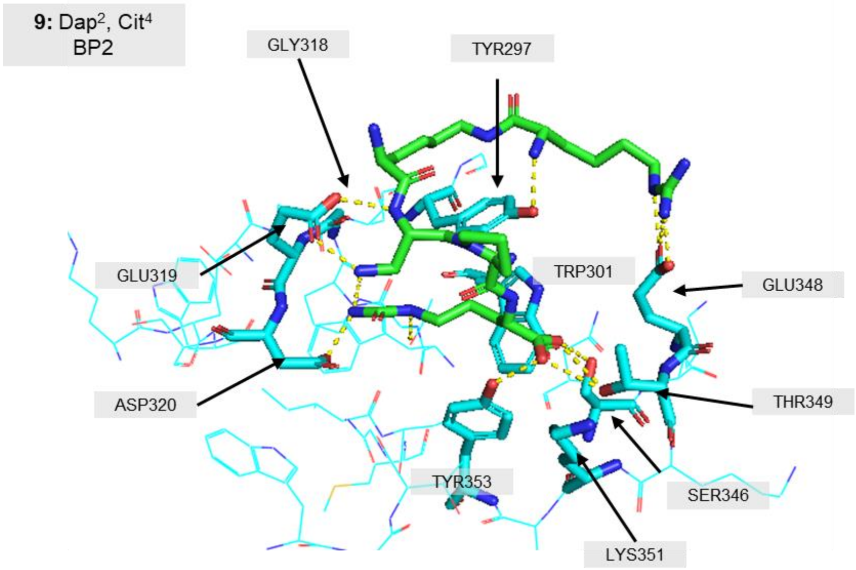

Protein in blue, ligand in green. Hydrogen display suppressed. Only selected residues of the protein are shown or labelled. Yellow dashed lines mark donor-acceptor pairs at the H-bonding distance (<3.2 Å)

Figure SI-19. Compound 10 modelled in Binding Pose 1 (BP1).

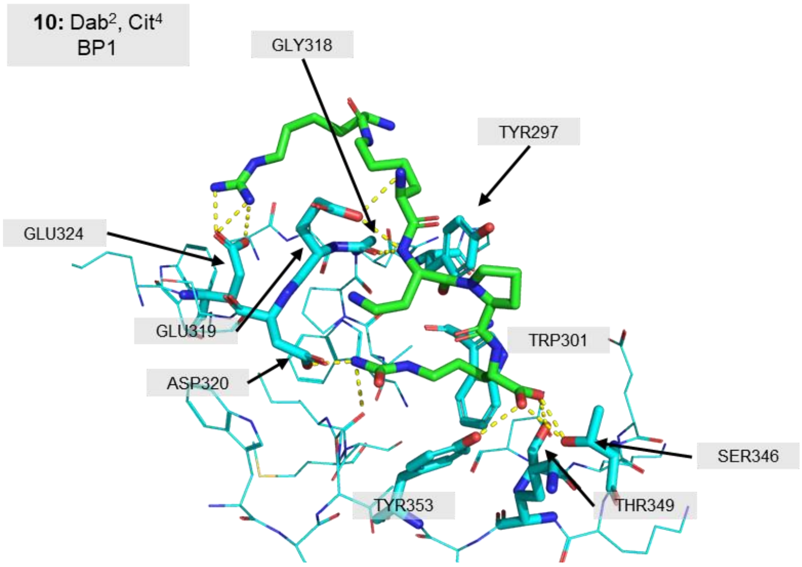

Protein in blue, ligand in green. Hydrogen display suppressed. Only selected residues of the protein are shown or labelled. Yellow dashed lines mark donor-acceptor pairs at the H-bonding distance (<3.2 Å)

Figure SI-20. Compound 10 modelled in Binding Pose 2 (BP2).

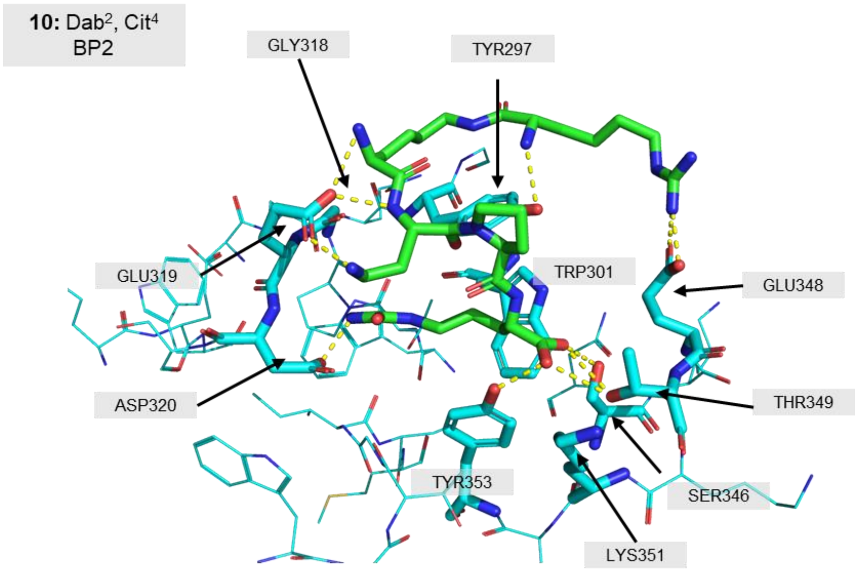

Protein in blue, ligand in green. Hydrogen display suppressed. Only selected residues of the protein are shown or labelled. Yellow dashed lines mark donor-acceptor pairs at the H-bonding distance (<3.2 Å)

Figure SI-21. Compound 11 modelled in Binding Pose 1 (BP1).

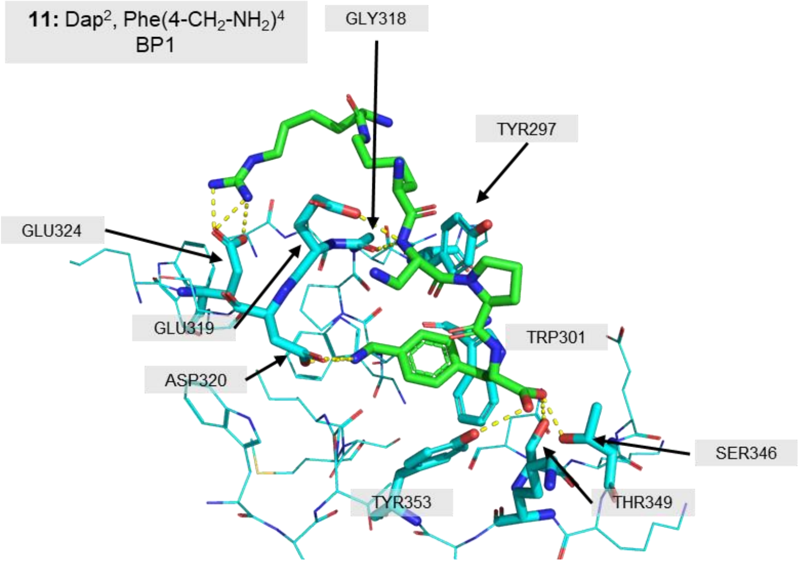

Protein in blue, ligand in green. Hydrogen display suppressed. Only selected residues of the protein are shown or labelled. Yellow dashed lines mark donor-acceptor pairs at the H-bonding distance (<3.2 Å)

Figure SI-22. Compound 11 modelled in Binding Pose 2 (BP2).

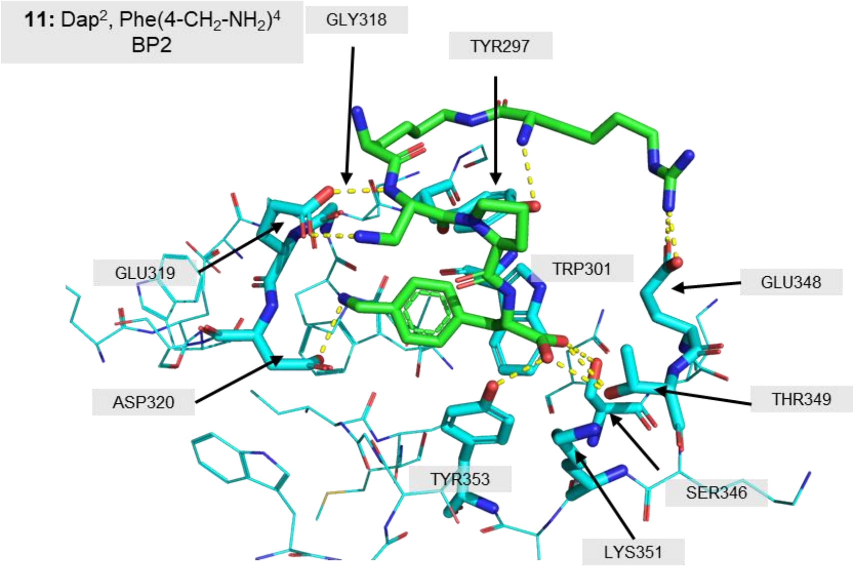

Protein in blue, ligand in green. Hydrogen display suppressed. Only selected residues of the protein are shown or labelled. Yellow dashed lines mark donor-acceptor pairs at the H-bonding distance (<3.2 Å)

Figure SI-23. Compound 12 modelled in Binding Pose 1 (BP1).

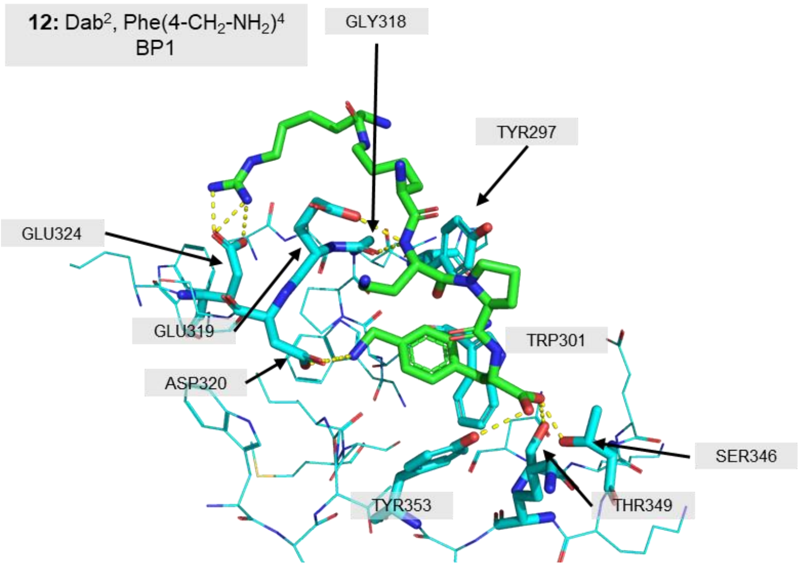

Protein in blue, ligand in green. Hydrogen display suppressed. Only selected residues of the protein are shown or labelled. Yellow dashed lines mark donor-acceptor pairs at the H-bonding distance (<3.2 Å)

Figure SI-24. Compound 12 modelled in Binding Pose 2 (BP2).

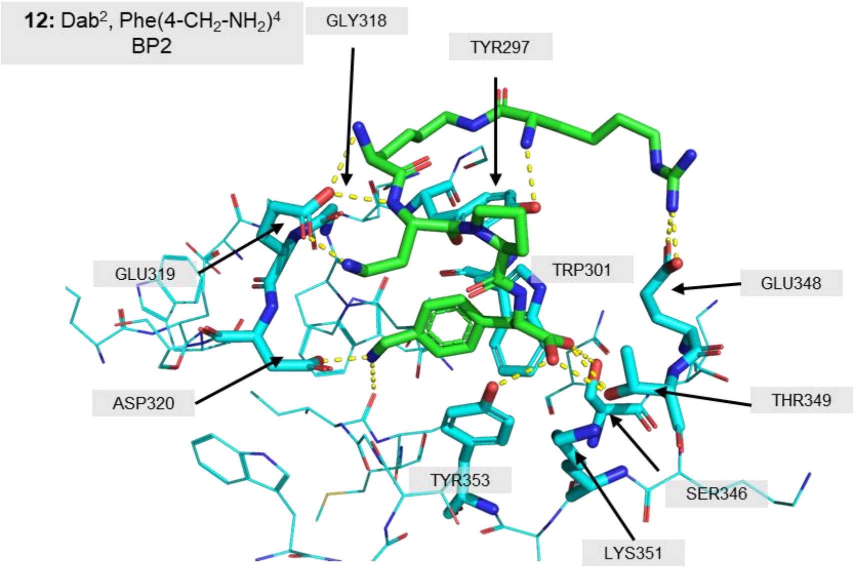

Protein in blue, ligand in green. Hydrogen display suppressed. Only selected residues of the protein are shown or labelled. Yellow dashed lines mark donor-acceptor pairs at the H-bonding distance (<3.2 Å)

Table SI-2: Physical Characteristics of peptidomimetics – **part 1**

No.	Peptidomimetic sequence	Molecular formula	HR-ESI-MS		
			Measured m/z $[M+H]^+$	Calculated m/z $[M+H]^+$	Diff. [ppm]
**1**	Lys(Har)-Dap-Pro-Arg	$C_{27}H_{53}N_{13}O_6$	656.43112	656.43145	-0.501
**2**	Lys(Har)-Dab-Pro-Arg	$C_{28}H_{55}N_{13}O_6$	670.44707	670.44710	-0.049
**3**	Lys(Har)-Dap-Pro-Har	$C_{28}H_{55}N_{13}O_6$	670.44718	670.44710	0.115
**4**	Lys(Har)-Dab-Pro-Har	$C_{29}H_{57}N_{13}O_6$	684.46246	684.46275	-0.428
**5**	Lys(Har)-Dap-Pro-Agb	$C_{26}H_{51}N_{13}O_6$	642.41600	642.41580	0.307
**6**	Lys(Har)-Dab-Pro-Agb	$C_{27}H_{53}N_{13}O_6$	656.43171	656.43145	0.392
**7**	Lys(Har)-Dap-Pro-Agp	$C_{25}H_{49}N_{13}O_6$	628.40059	628.40015	0.696
**8**	Lys(Har)-Dab-Pro-Agp	$C_{26}H_{51}N_{13}O_6$	642.41622	642.41580	0.649
**9**	Lys(Har)-Dap-Pro-Cit	$C_{27}H_{52}N_{12}O_7$	657.41555	657.41547	0.124
**10**	Lys(Har)-Dab-Pro-Cit	$C_{28}H_{54}N_{12}O_7$	671.43159	671.43112	0.702
**11**	Lys(Har)-Dap-Pro-Phe(4-CH_2-NH_2)	$C_{31}H_{53}N_{11}O_6$	676.42566	676.42530	0.525
**12**	Lys(Har)-Dab-Pro-Phe(4-CH_2-NH_2)	$C_{32}H_{55}N_{11}O_6$	690.44126	690.44095	0.442

Table SI-3: Physical Characteristics of peptidomimetics – **part 2**

No.	Peptidomimetic sequence	HPLC ^{t}R(min)*	% Purity
**1**	Lys(Har)-Dap-Pro-Arg	13.908	99.4 %
**2**	Lys(Har)-Dab-Pro-Arg	13.383	99.3 %
**3**	Lys(Har)-Dap-Pro-Har	14.965	98.7 %
**4**	Lys(Har)-Dab-Pro-Har	14.237	99.0 %
**5**	Lys(Har)-Dap-Pro-Agb	13.305	99.1 %
**6**	Lys(Har)-Dab-Pro-Agb	12.092	98.4 %
**7**	Lys(Har)-Dap-Pro-Agp	12.695	98.7 %
**8**	Lys(Har)-Dab-Pro-Agp	11.845	98.3 %
**9**	Lys(Har)-Dap-Pro-Cit	13.736	98.6 %
**10**	Lys(Har)-Dab-Pro-Cit	12.896	98.8 %
**11**	Lys(Har)-Dap-Pro-Phe(4-CH_2-NH_2)	15.483	98.4 %
**12**	Lys(Har)-Dab-Pro-Phe(4-CH_2-NH_2)	15.026	98.0 %

*Non-linear gradient was applied: 0–15% B for 20 min followed by an increase to 70% B from 20 to 26 min

Figure SI-25: Analytical RP-HPLC traces (absorbance at 215 nm) of all peptidomimetics **1-12.**
Lys(Har)-Dap-Pro-Arg (**1**)

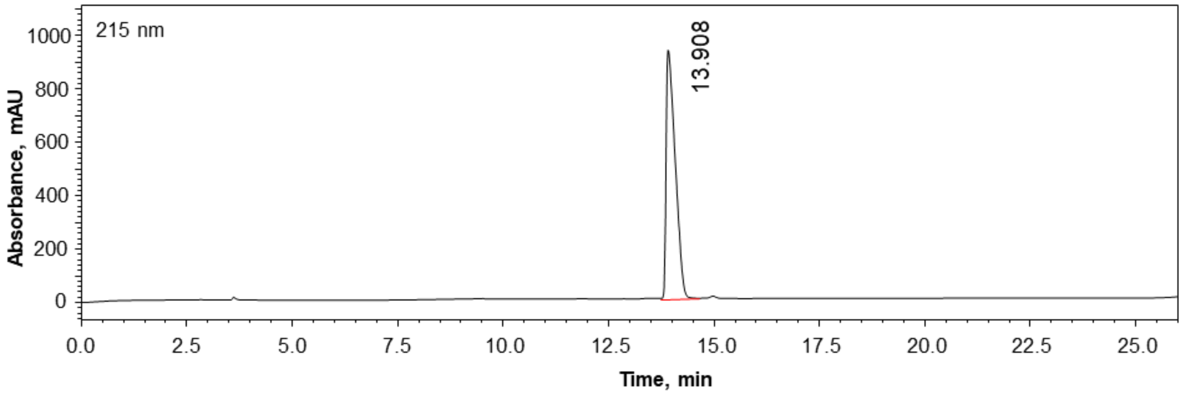

Lys(Har)-Dab-Pro-Arg (**2**)

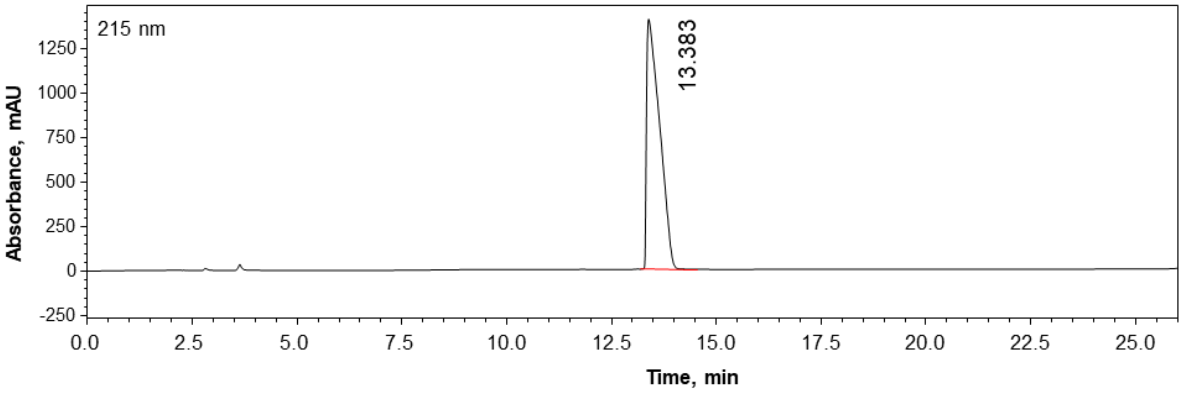

Lys(Har)-Dap-Pro-Har (**3**)

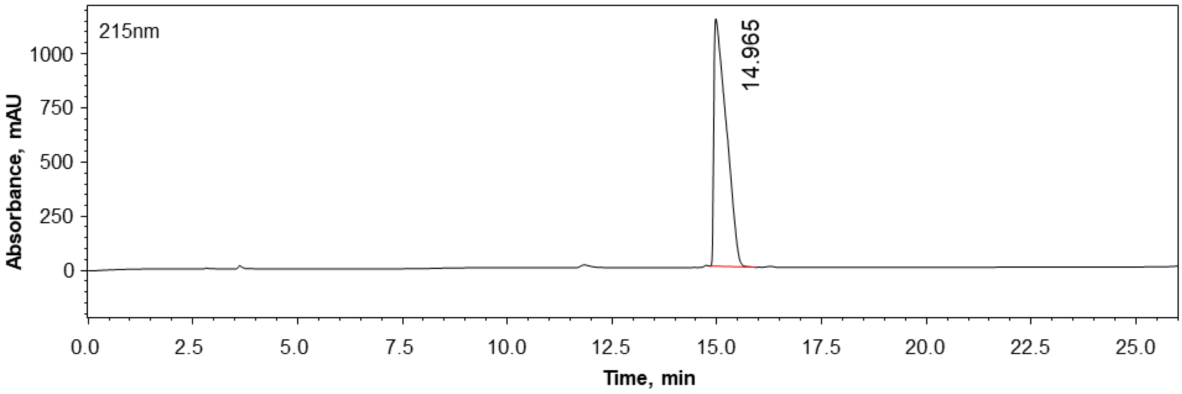

Lys(Har)-Dab-Pro-Har (**4**)

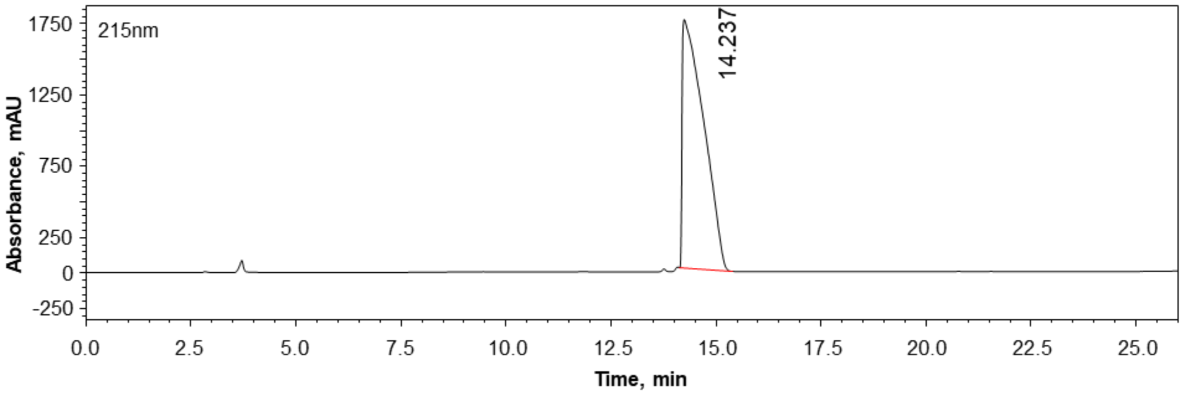

Lys(Har)-Dap-Pro-Agb (**5**)

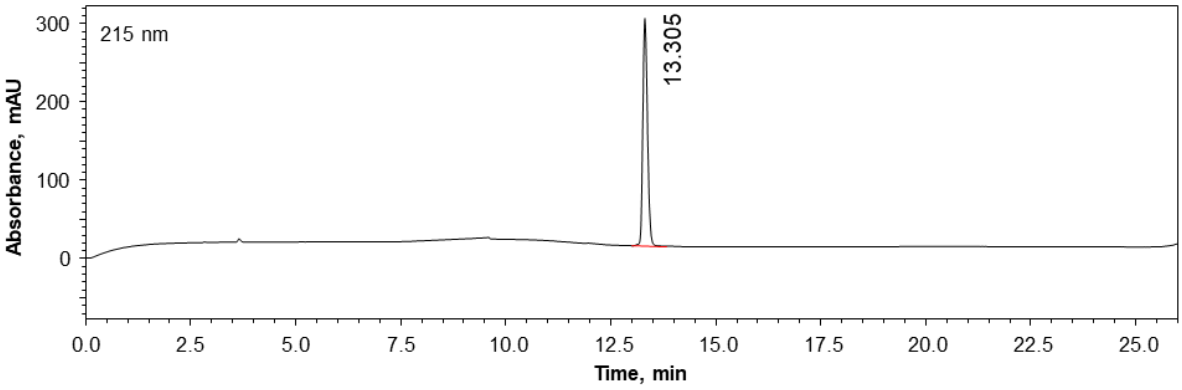

Lys(Har)-Dab-Pro-Agb (**6**)

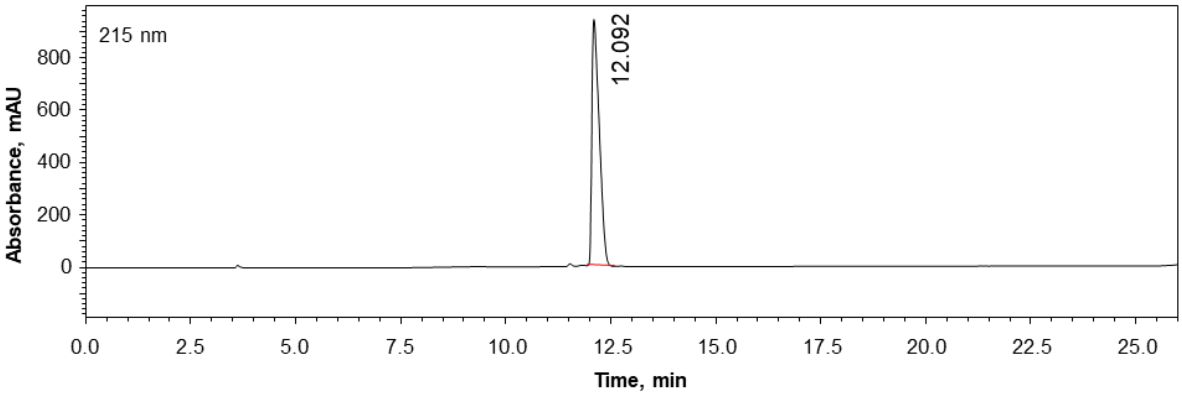

Lys(Har)-Dap-Pro-Agp (**7**)

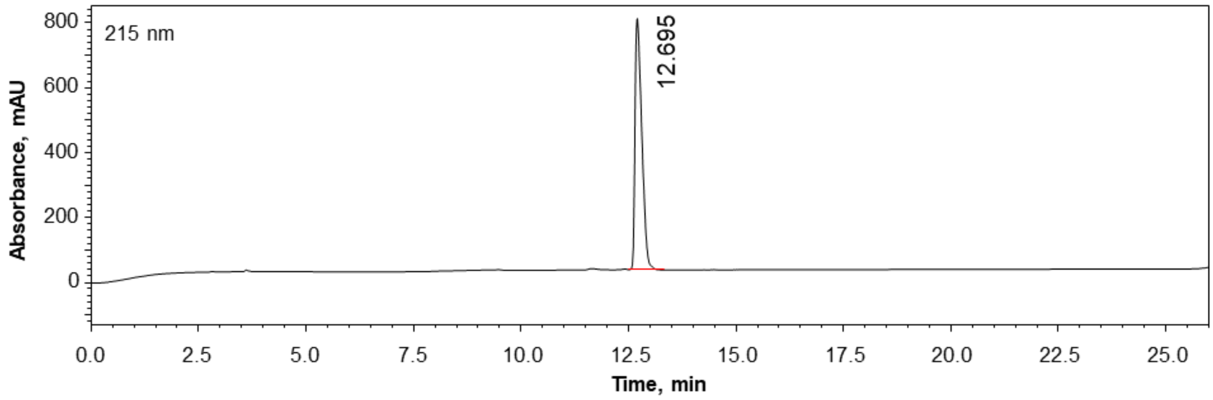

Lys(Har)-Dab-Pro-Agp (**8**)

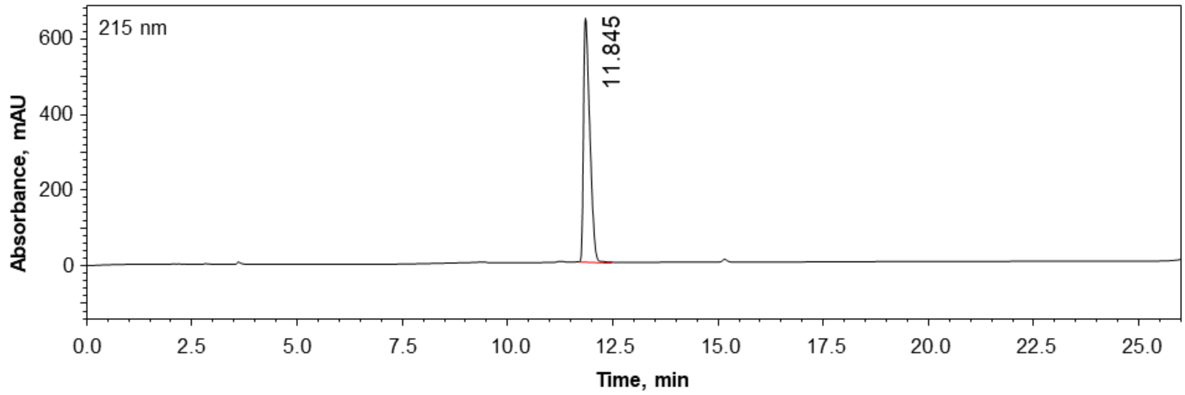

Lys(Har)-Dap-Pro-Cit (**9**)

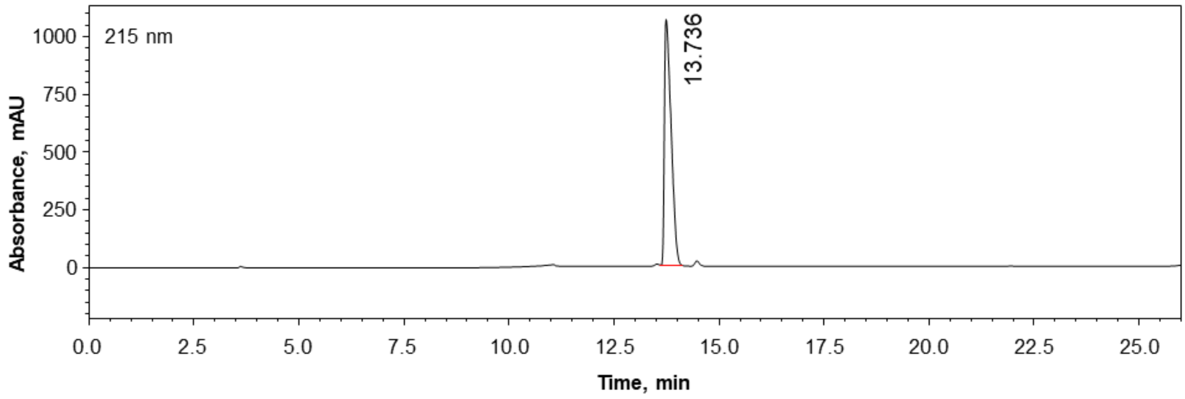

Lys(Har)-Dab-Pro-Cit (**10**)

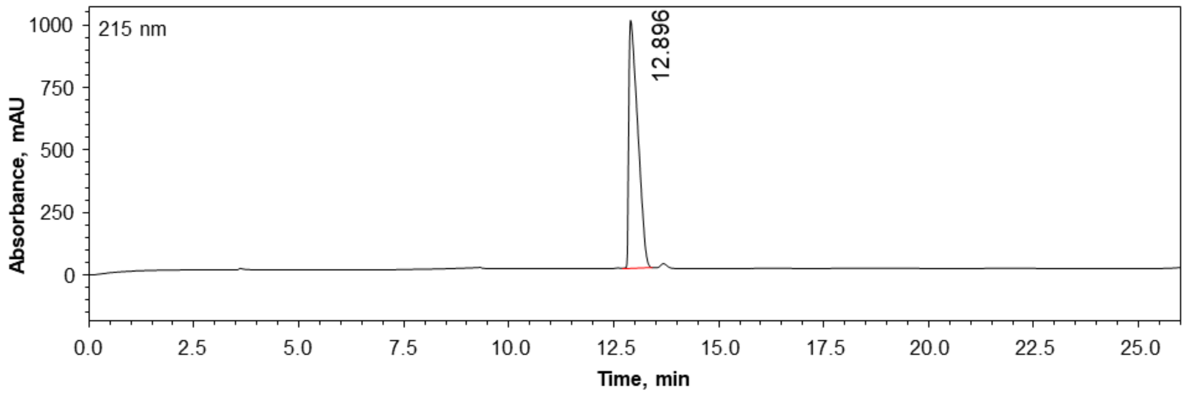

Lys(Har)-Dap-Pro-Phe(4-CH_2-NH_2) (**11**)

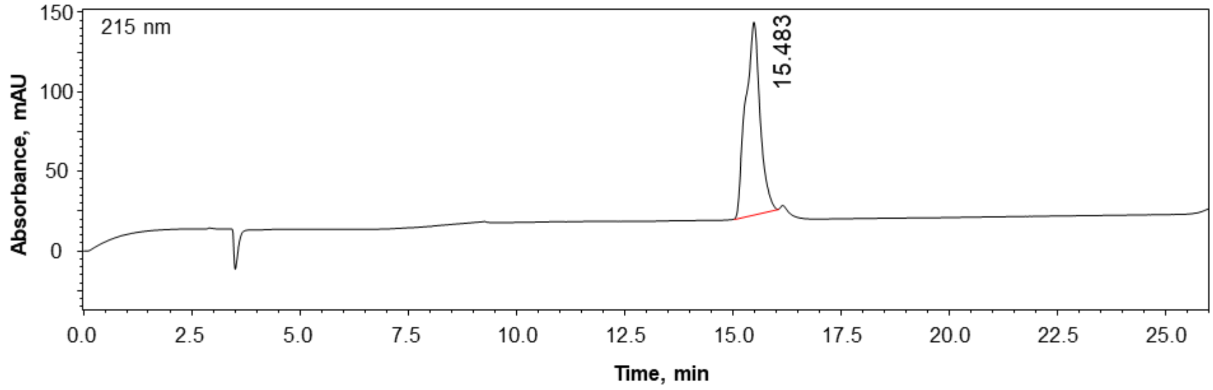

Lys(Har)-Dab-Pro-Phe(4-CH_2-NH_2) (**12**)

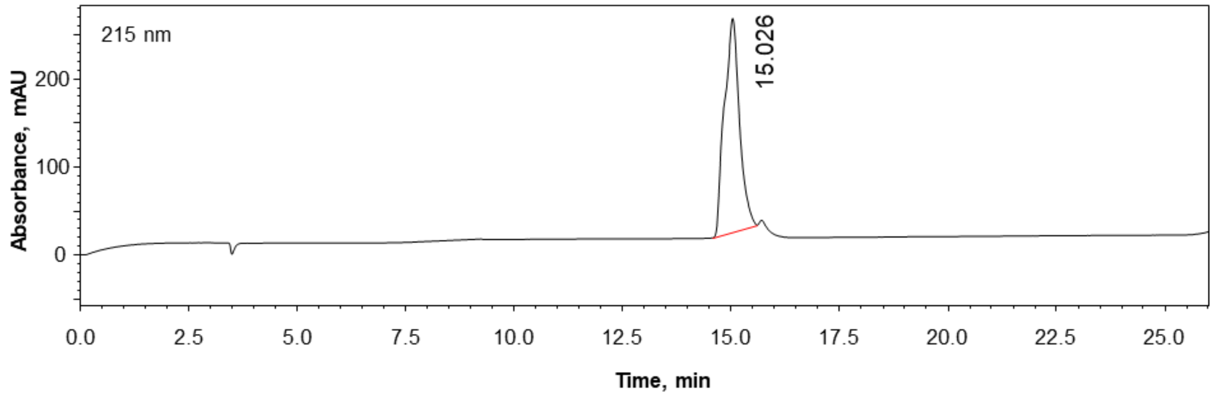

Figure SI-26: RP-HPLC traces and LC-MS results recorded for selected time points during stability tests of Lys(Har)-Dap-Pro-Arg (**1**).

RP-HPLC trace at 0 minute:

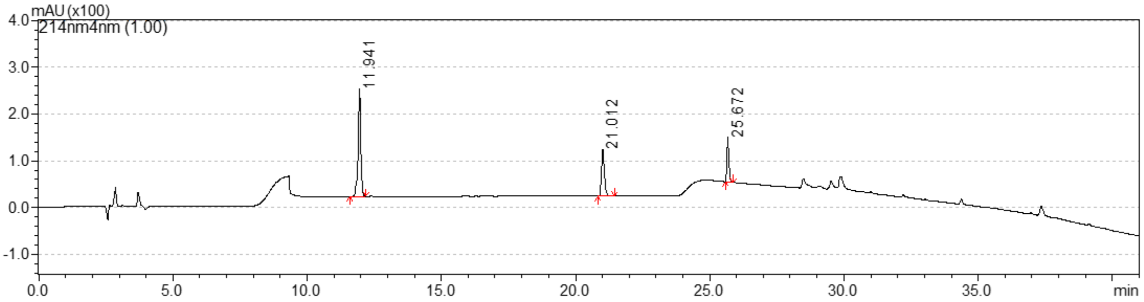

Peak at ^{t}R = 11.941 min; Lys(Har)-Dap-Pro-Arg (**1**)
Peak at ^{t}R = 21.012 min; internal standard (H-Trp-OH)
Peak at ^{t}R = 25.672 min; internal standard (Z-Lys-OH)

RP-HPLC traces (absorbance at 215 nm), recorded for selected time points (0 minute, 24 h, 48 h and 72 h):

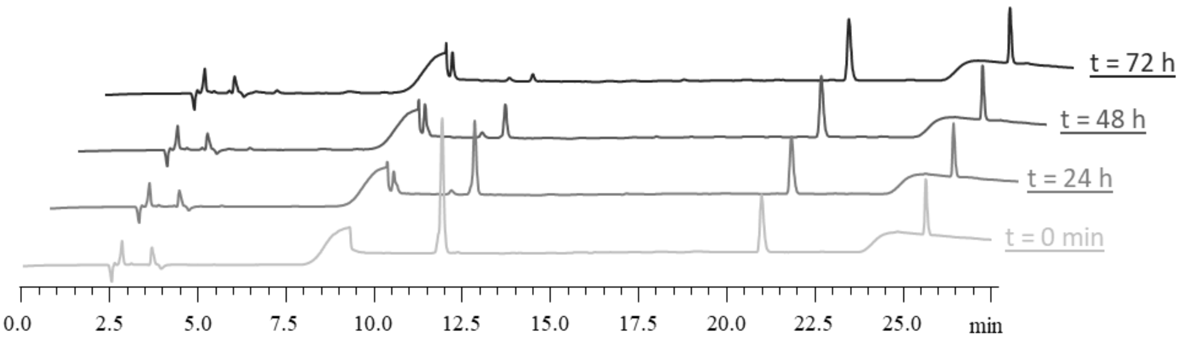

TIC (total ion current) MS-chromatogram, recorded for selected time points (0 minute, 24 h). Extracted time of analysis: 0.0 - 30.0 min:

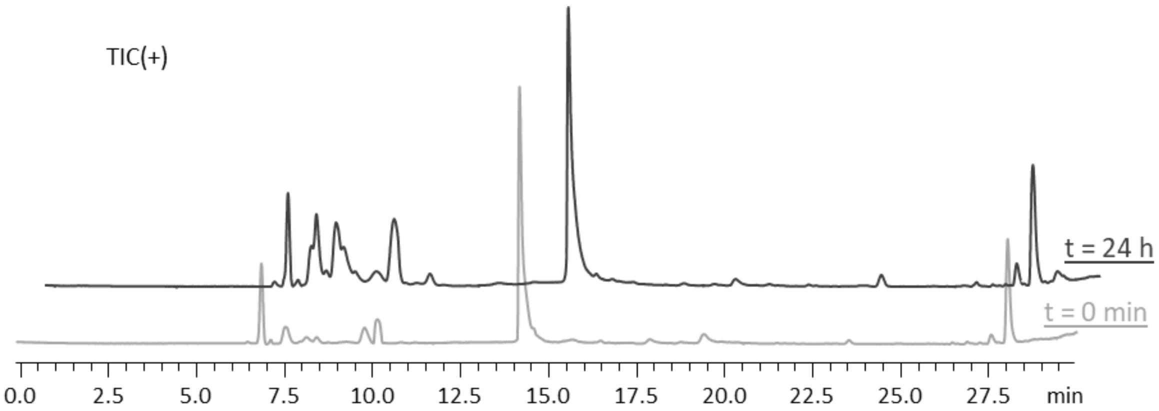

MS results:

Cmpd	Measured HR-ESI-MS	Calculated	Diff. (mDa)
**Lys(Har)-Dap-Pro-Arg**	219.4820 $[M+3H]^{3+}$<br>328.7194 $[M+2H]^{2+}$<br>656.4309 $[M+H]^{+}$	219.4825 $[M+3H]^{3+}$<br>328.7199 $[M+2H]^{2+}$<br>656.4320 $[M+H]^{+}$	-0.5<br>-0.5<br>-1.1
**Lys(Har)-Dap**	403.2769 $[M+H]^{+}$<br>425.2588 $[M+Na]^{+}$	403.2781 $[M+H]^{+}$<br>425.2601 $[M+Na]^{+}$	-1.2<br>-1.3
**Lys(Har)**	317.2292 $[M+H]^{+}$	317.2301 $[M+H]^{+}$	-0.9
**Lys-Dap-Pro-Arg**	243.6608 $[M+2H]^{2+}$<br>486.3140 $[M+H]^{+}$<br>971.6210 $[2M+H]^{+}$	243.6615 $[M+2H]^{2+}$<br>486.3152 $[M+H]^{+}$<br>971.6226 $[2M+H]^{+}$	-0.7<br>-1.2<br>-1.6
**Pro**	116.0703 $[M+H]^{+}$<br>231.1335 $[2M+H]^{+}$	116.0711 $[M+H]^{+}$<br>231.1344 $[2M+H]^{+}$	-0.8<br>-0.9
**Arg**	175.1187 $[M+H]^{+}$<br>349.2300 $[2M+H]^{+}$	175.1195 $[M+H]^{+}$<br>349.2312 $[2M+H]^{+}$	-0.8<br>-1.2
**Har**	189.1343 $[M+H]^{+}$<br>377.2613 $[2M+H]^{+}$	189.1351 $[M+H]^{+}$<br>377.2624 $[2M+H]^{+}$	-0.8<br>-1.1

Figure SI-27: RP-HPLC traces and LC-MS results recorded for selected time points during stability tests of Lys(Har)-Dab-Pro-Arg (**2**).

RP-HPLC trace at 0 minute:

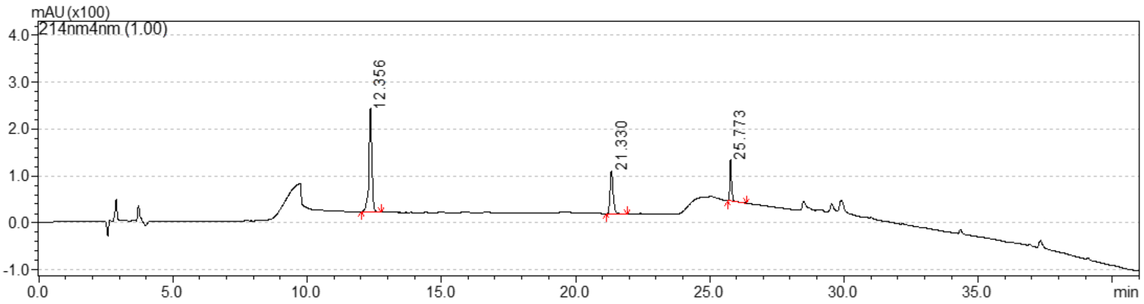

Peak at ^{t}R = 12.356 min; Lys(Har)-Dab-Pro-Arg (**2**)
Peak at ^{t}R = 21.330 min; internal standard (H-Trp-OH)
Peak at ^{t}R = 25.773 min; internal standard (Z-Lys-OH)

RP-HPLC traces (absorbance at 215 nm), recorded for selected time points (0 minute, 24 h, 48 h and 72 h):

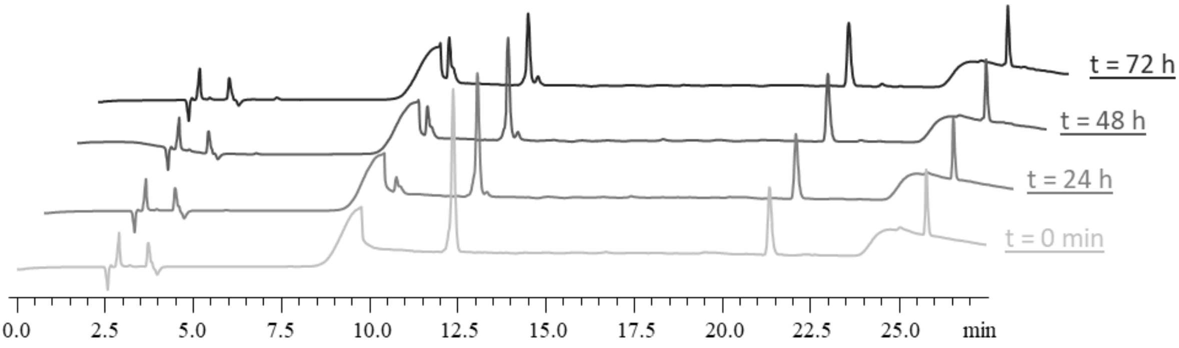

TIC (total ion current) MS-chromatogram, recorded for selected time points (0 minute, 72 h). Extracted time of analysis: 0.0 - 30.0 min:

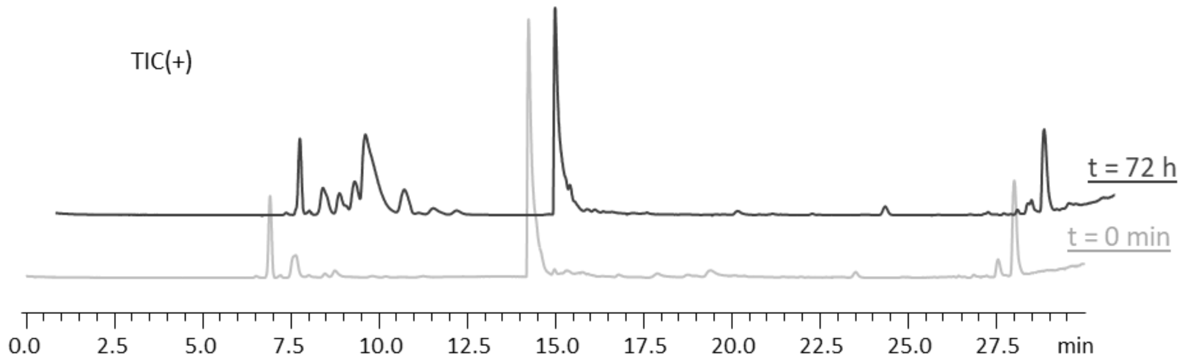

MS results:

Cmpd	Measured HR-ESI-MS	Calculated	Diff. (mDa)

**Lys(Har)-Dab-Pro-Arg**	224.1534 $[M+3H]^{3+}$<br>335.7264 $[M+2H]^{2+}$<br>670.4452 $[M+H]^{+}$	224.1544 $[M+3H]^{3+}$<br>335.7277 $[M+2H]^{2+}$<br>670.4476 $[M+H]^{+}$	-1.0<br>-1.3<br>-2.4
**Lys(Har)-Dab**	209.1498 $[M+2H]^{2+}$<br>417.2921 $[M+H]^{+}$	209.1508 $[M+2H]^{2+}$<br>417.2937 $[M+H]^{+}$	-1.0<br>-1.6
**Lys(Har)**	317.2285 $[M+H]^{+}$	317.2301 $[M+H]^{+}$	-1.6
**Lys-Dab-Pro-Arg**	250.6682 $[M+2H]^{2+}$<br>500.3288 $[M+H]^{+}$<br>522.3107 $[M+Na]^{+}$	250.6693 $[M+2H]^{2+}$<br>500.3309 $[M+H]^{+}$<br>522.3128 $[M+Na]^{+}$	-1.1<br>-2.1<br>-2.1
**Lys-Dab-Pro**	344.2283 $[M+H]^{+}$<br>366.2102 $[M+Na]^{+}$	344.2298 $[M+H]^{+}$<br>366.2117 $[M+Na]^{+}$	-1.5<br>-1.5
**Pro**	116.0701 $[M+H]^{+}$	116.0711 $[M+H]^{+}$	-1.0
**Arg**	175.1183 $[M+H]^{+}$<br>349.2295 $[2M+H]^{+}$	175.1195 $[M+H]^{+}$<br>349.2312 $[2M+H]^{+}$	-1.2<br>-1.7
**Har**	189.1339 $[M+H]^{+}$<br>377.2607 $[2M+H]^{+}$	189.1351 $[M+H]^{+}$<br>377.2624 $[2M+H]^{+}$	-1.2<br>-1.7

Figure SI-28: RP-HPLC traces and LC-MS results recorded for selected time points during stability tests of Lys(Har)-Dap-Pro-Har (**3**).

RP-HPLC trace at 0 minute:

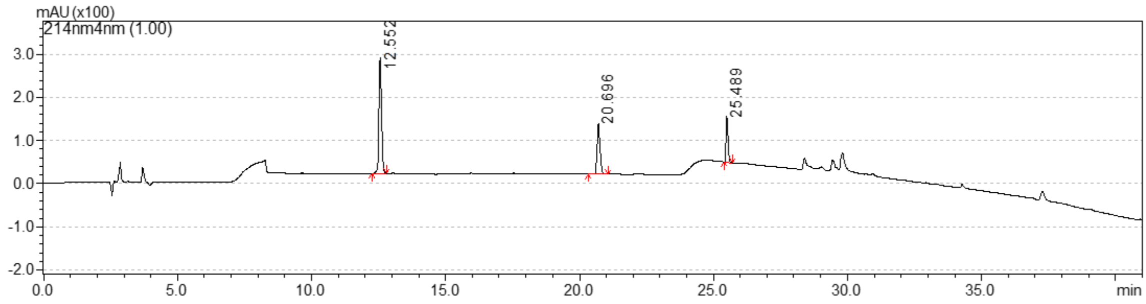

Peak at ^{t}R = 12.552 min; Lys(Har)-Dap-Pro-Har (**3**)
Peak at ^{t}R = 20.696 min; internal standard (H-Trp-OH)
Peak at ^{t}R = 25.489 min; internal standard (Z-Lys-OH)

RP-HPLC traces (absorbance at 215 nm), recorded for selected time points (0 minute, 24 h, 48 h and 72 h):

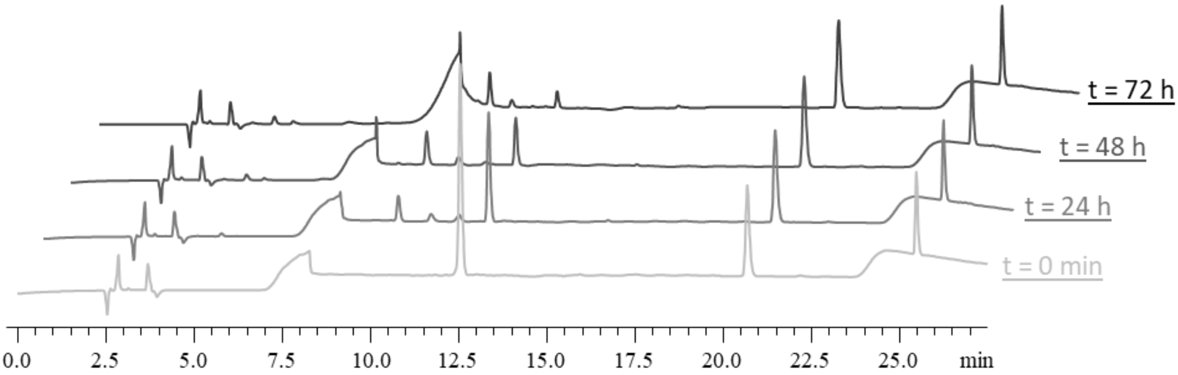

TIC (total ion current) MS-chromatogram, recorded for selected time points (0 minute, 24 h). Extracted time of analysis: 0.0 - 30.0 min:

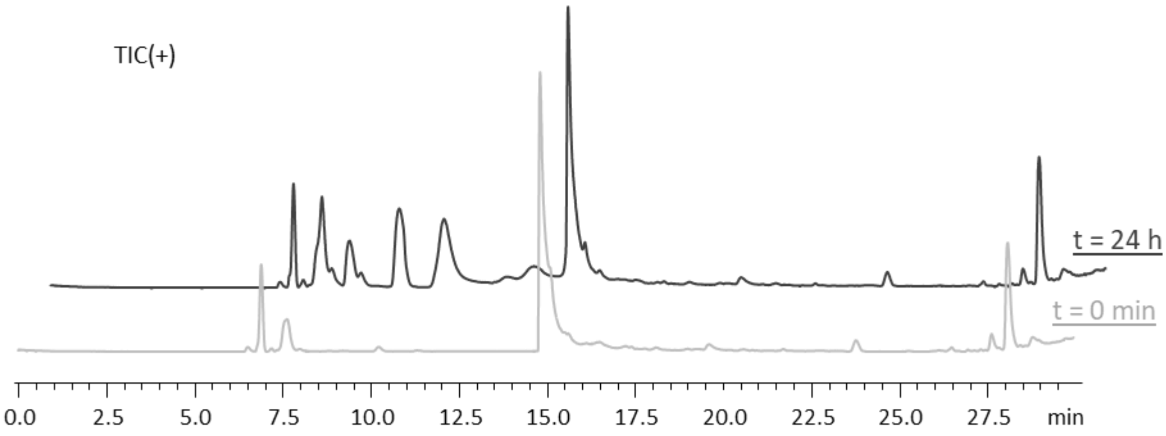

MS results:

Cmpd	Measured HR-ESI-MS	Calculated	Diff. (mDa)
**Lys(Har)-Dap-Pro-Har**	224.1539 $[M+3H]^{3+}$<br>335.7270 $[M+2H]^{2+}$<br>670.4463 $[M+H]^{+}$	224.1544 $[M+3H]^{3+}$<br>335.7277 $[M+2H]^{2+}$<br>670.4476 $[M+H]^{+}$	-0.5<br>-0.7<br>1.3
**Lys(Har)-Dap**	403.2770 $[M+H]^{+}$<br>425.2588 $[M+Na]^{+}$	403.2781 $[M+H]^{+}$<br>425.2601 $[M+Na]^{+}$	-1.1<br>-1.3
**Lys(Har)**	317.2291 $[M+H]^{+}$<br>633.4511 $[2M+H]^{+}$	317.2301 $[M+H]^{+}$<br>633.4524 $[2M+H]^{+}$	-1.0<br>-1.3
**Lys-Dap-Pro-Har**	250.6685 $[M+2H]^{2+}$<br>500.3295 $[M+H]^{+}$<br>999.6530 $[2M+H]^{+}$	250.6694 $[M+2H]^{2+}$<br>500.3309 $[M+H]^{+}$<br>999.6539 $[2M+H]^{+}$	-0.9<br>-1.4<br>-0.9
**Lys-Dap-Pro**	330.2129 $[M+H]^{+}$<br>352.1948 $[M+Na]^{+}$	330.2141 $[M+H]^{+}$<br>352.1961 $[M+Na]^{+}$	-1.2<br>-1.3
**Pro**	116.0703 $[M+H]^{+}$	116.0711 $[M+H]^{+}$	-0.8
**Har**	189.1344 $[M+H]^{+}$<br>377.2616 $[2M+H]^{+}$	189.1351 $[M+H]^{+}$<br>377.2624 $[2M+H]^{+}$	-0.7<br>-0.8

Figure SI-29: RP-HPLC traces and LC-MS results recorded for selected time points during stability tests of Lys(Har)-Dab-Pro-Har (**4**).

RP-HPLC trace at 0 minute:

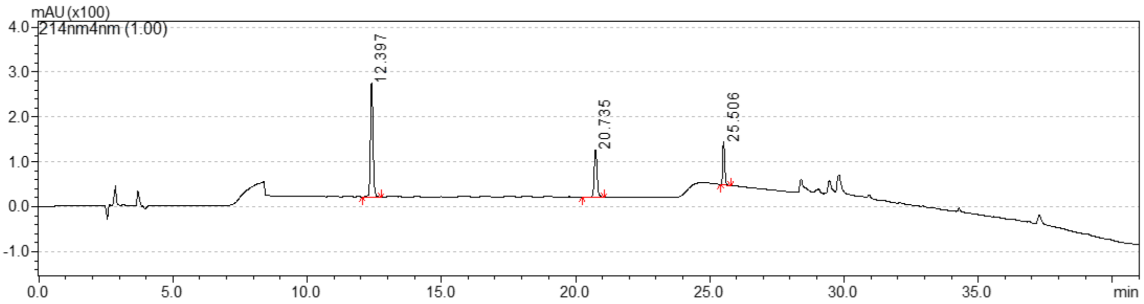

Peak at ^{t}R = 12.397 min; Lys(Har)-Dab-Pro-Har (**4**)
Peak at ^{t}R = 20.735 min; internal standard (H-Trp-OH)
Peak at ^{t}R = 25.506 min; internal standard (Z-Lys-OH)

RP-HPLC traces (absorbance at 215 nm), recorded for selected time points (0 minute, 24 h, 48 h and 72 h):

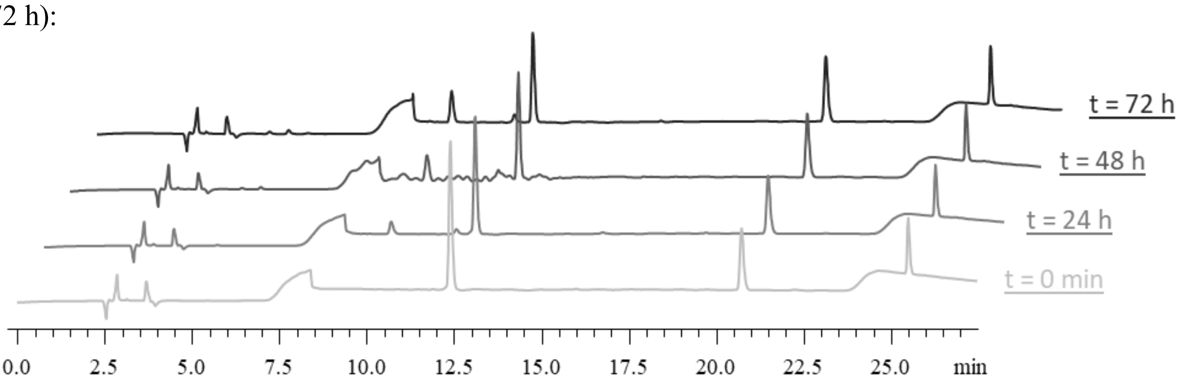

TIC (total ion current) MS-chromatogram, recorded for selected time points (0 minute, 72 h). Extracted time of analysis: 0.0 - 30.0 min:

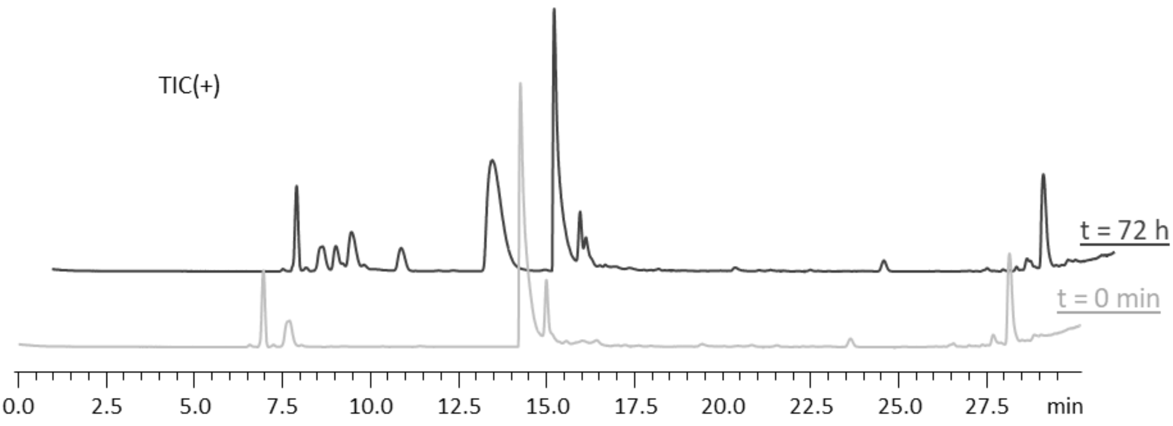

MS results:

Cmpd	Measured HR-ESI-MS	Calculated	Diff. (mDa)

**Lys(Har)-Dab-Pro-Har**	228.8255 $[M+3H]^{3+}$<br>342.7345 $[M+2H]^{2+}$<br>684.4613 $[M+H]^{+}$	228.8263 $[M+3H]^{3+}$<br>342.7356 $[M+2H]^{2+}$<br>684.4633 $[M+H]^{+}$	-0.8<br>-1.1<br>-2.0
**Lys(Har)-Dab**	209.1498 $[M+2H]^{2+}$<br>417.2921 $[M+H]^{+}$	209.1508 $[M+2H]^{2+}$<br>417.2937 $[M+H]^{+}$	-1.0<br>-1.6
**Lys(Har)**	317.2286 $[M+H]^{+}$	317.2301 $[M+H]^{+}$	-1.5
**Lys-Dab-Pro-Har**	257.6762 $[M+2H]^{2+}$<br>514.3448 $[M+H]^{+}$<br>1027.6827 $[2M+H]^{+}$	257.6772 $[M+2H]^{2+}$<br>514.3465 $[M+H]^{+}$<br>1027.6852 $[2M+H]^{+}$	-1.0<br>-1.7<br>-2.5
**Lys-Dab-Pro**	344.2283 $[M+H]^{+}$<br>366.2103 $[M+Na]^{+}$	344.2298 $[M+H]^{+}$<br>366.2117 $[M+Na]^{+}$	-1.5<br>-1.4
**Pro**	116.0701 $[M+H]^{+}$	116.0711 $[M+H]^{+}$	-1.0
**Har**	189.1340 $[M+H]^{+}$<br>377.2609 $[2M+H]^{+}$	189.1351 $[M+H]^{+}$<br>377.2624 $[2M+H]^{+}$	-1.1<br>-1.5

Figure SI-30: RP-HPLC traces and LC-MS results recorded for selected time points during stability tests of Lys(Har)-Dap-Pro-Phe(4-CH_2-NH_2) (**11**).

RP-HPLC trace at 0 minute:

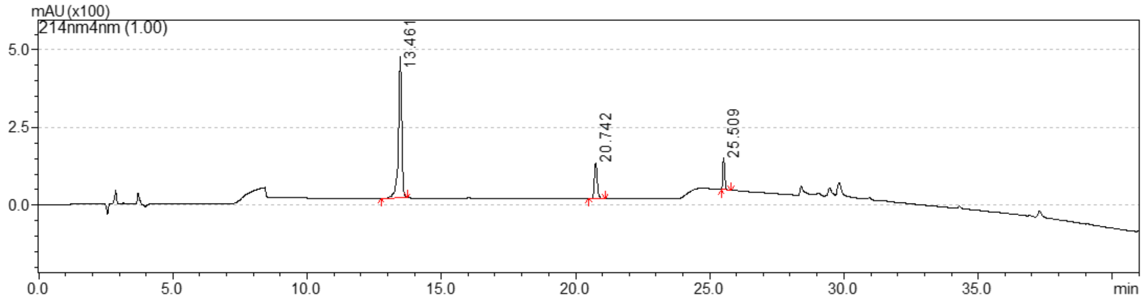

Peak at ^{t}R = 13.461 min; Lys(Har)-Dap-Pro-Phe(4-CH_2-NH_2) (**11**)
Peak at ^{t}R = 20.742 min; internal standard (H-Trp-OH)
Peak at ^{t}R = 25.509 min; internal standard (Z-Lys-OH)

RP-HPLC traces (absorbance at 215 nm), recorded for selected time points (0 minute, 24 h, 48 h and 72 h):

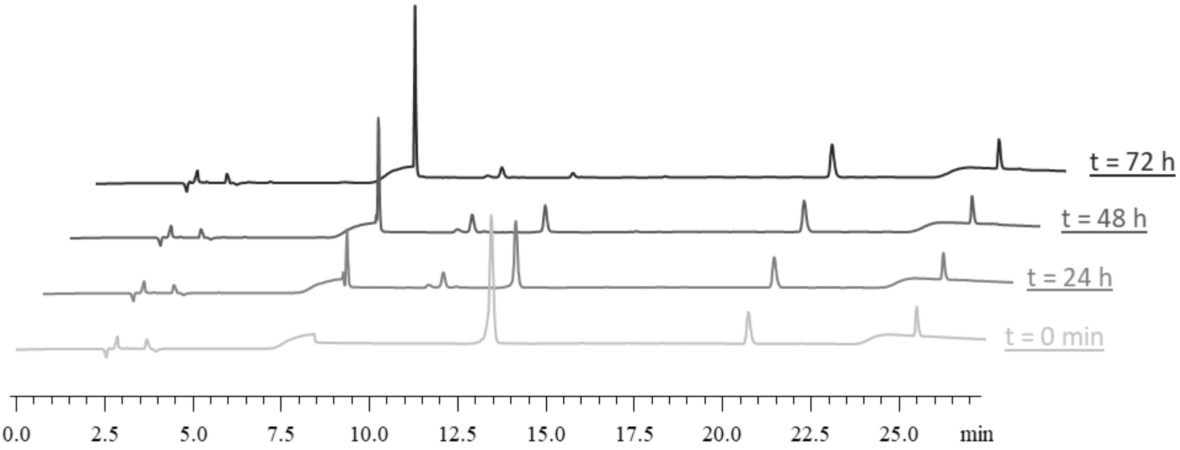

TIC (total ion current) MS-chromatogram, recorded for selected time points (0 minute, 24 h).
Extracted time of analysis: 0.0 - 30.0 min:

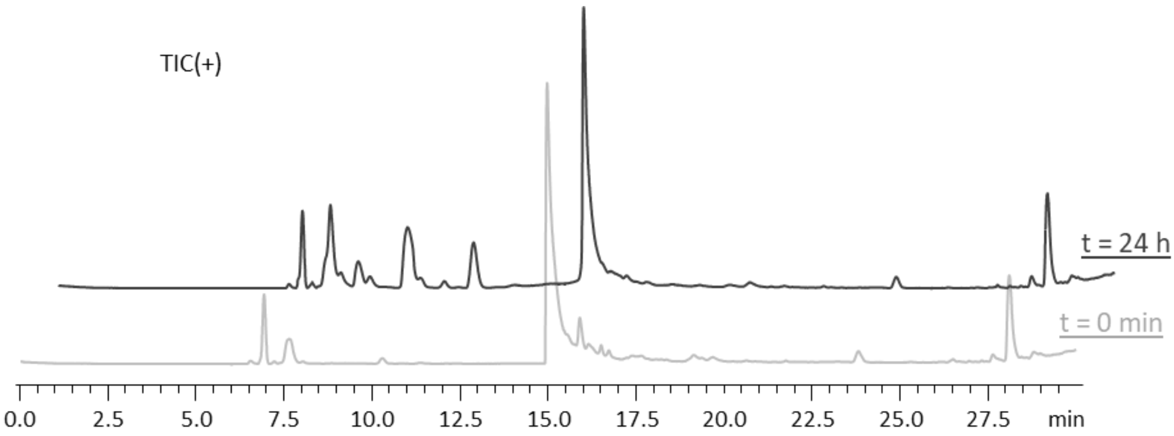

MS results:

Cmpd	Measured HR-ESI-MS	Calculated	Diff. (mDa)

**Lys(Har)-Dap-Pro-Phe(4-CH_2-NH_2)**	338.7159 $[M+2H]^{2+}$<br>676.4241 $[M+H]^{+}$	338.7168 $[M+2H]^{2+}$<br>676.4258 $[M+H]^{+}$	-0.9<br>-1.7
**Lys(Har)-Dap**	403.2767 $[M+H]^{+}$<br>425.2585 $[M+Na]^{+}$	403.2781 $[M+H]^{+}$<br>425.2601 $[M+Na]^{+}$	-1.4<br>-1.6
**Lys(Har)**	317.2290 $[M+H]^{+}$<br>633.4507 $[2M+H]^{+}$	317.2301 $[M+H]^{+}$<br>633.4524 $[2M+H]^{+}$	-1.1<br>-1.7
**Phe(4-CH_2-NH_2)**	195.1121 $[M+H]^{+}$<br>389.2173 $[2M+H]^{+}$	195.1133 $[M+H]^{+}$<br>389.2189 $[2M+H]^{+}$	-1.2<br>-1.6
**Lys-Dap-Pro**	330.2127 $[M+H]^{+}$<br>352.1946 $[M+Na]^{+}$	330.2141 $[M+H]^{+}$<br>352.1961 $[M+Na]^{+}$	-1.4<br>-1.5
**Pro**	116.0701 $[M+H]^{+}$	116.0711 $[M+H]^{+}$	-1.0
**Har**	189.1340 $[M+H]^{+}$	189.1351 $[M+H]^{+}$	-1.1

Figure SI-31: RP-HPLC traces and MS results recorded for selected time points during stability tests of Lys(Har)-Dab-Pro-Phe(4-CH_2-NH_2) (**12**).

RP-HPLC trace at 0 minute:

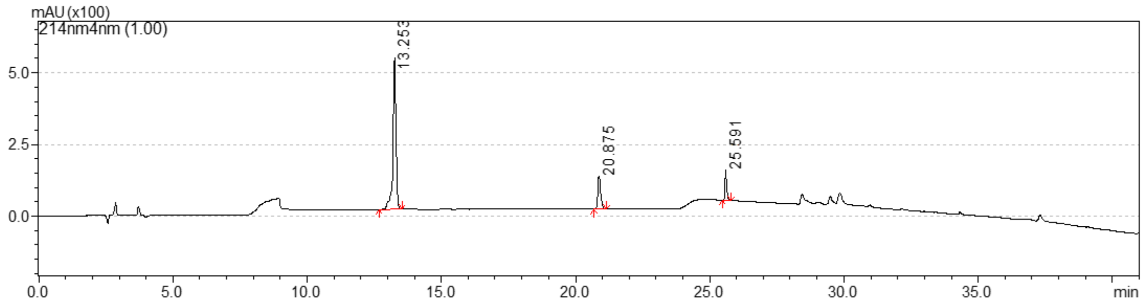

Peak at ^{t}R = 13.253 min; Lys(Har)-Dab-Pro-Phe(4-CH_2-NH_2) (**12**)
Peak at ^{t}R = 20.875 min; internal standard (H-Trp-OH)
Peak at ^{t}R = 25.591 min; internal standard (Z-Lys-OH)

RP-HPLC traces (absorbance at 215 nm), recorded for selected time points (0 minute, 24 h, 48 h and 72 h):

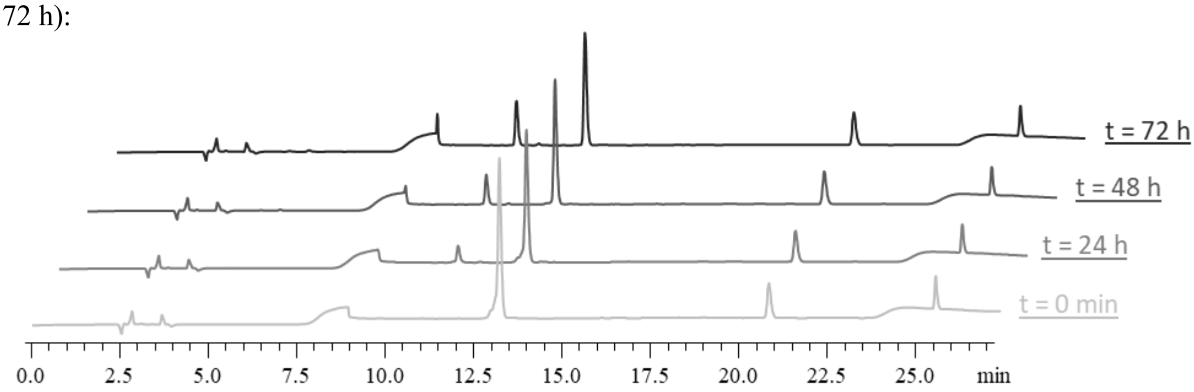

TIC (total ion current) MS-chromatogram, recorded for selected time points (0 minute, 72 h). Extracted time of analysis: 0.0 - 30.0 min:

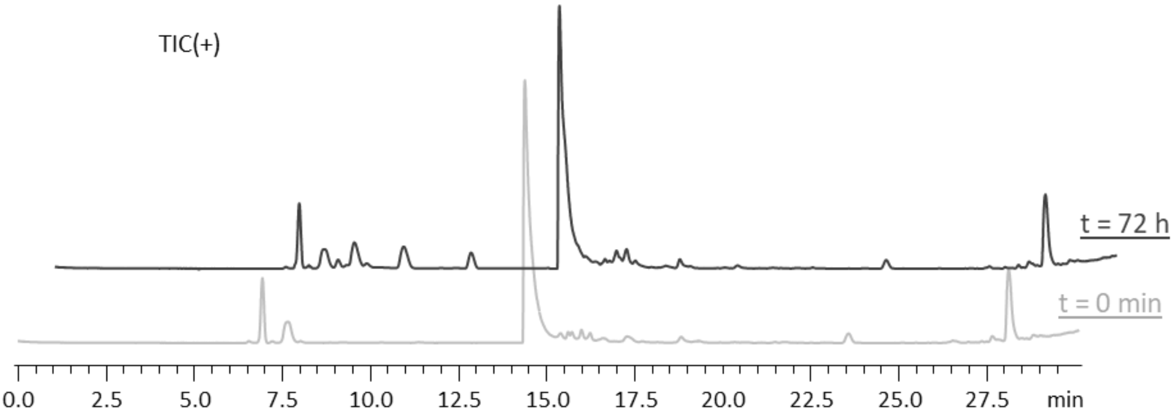

MS results:

Cmpd	Measured HR-ESI-MS	Calculated	Diff. (mDa)

**Lys(Har)-Dab-Pro-Phe(4-CH_2-NH_2)**	345.7237 $[M+2H]^{2+}$<br>690.4398 $[M+H]^+$	345.7247 $[M+2H]^{2+}$<br>690.4415 $[M+H]^+$	-1.0<br>-1.7
**Lys-Dab-Pro-Phe(4-CH_2-NH_2)**	260.6654 $[M+2H]^{2+}$<br>520.3234 $[M+H]^+$	260.6663 $[M+2H]^{2+}$<br>520.3247 $[M+H]^+$	-0.9<br>-1.3
**Lys(Har)-Dab**	209.1499 $[M+2H]^{2+}$<br>417.2924 $[M+H]^+$	209.1508 $[M+2H]^{2+}$<br>417.2937 $[M+H]^+$	-0.9<br>-1.3
**Lys-Dab-Pro**	344.2287 $[M+H]^+$<br>366.2101 $[M+Na]^+$	344.2298 $[M+H]^+$<br>366.2117 $[M+Na]^+$	-1.1<br>-1.6
**Lys(Har)**	317.2288 $[M+H]^+$	317.2301 $[M+H]^+$	-1.3
**Phe(4-CH_2-NH_2)**	195.1122 $[M+H]^+$<br>389.2175 $[2M+H]^+$	195.1133 $[M+H]^+$<br>389.2189 $[2M+H]^+$	-1.1<br>-1.4
**Pro**	116.0702 $[M+H]^+$	116.0711 $[M+H]^+$	-0.9
**Har**	189.1341 $[M+H]^+$<br>377.2611 $[2M+H]^+$	189.1351 $[M+H]^+$<br>377.2625 $[2M+H]^+$	-1.0<br>-1.4

Figure SI-32: RP-HPLC traces recorded for selected time points during serum activity tests with Tyr-Pro-Phe-Phe-NH_2 (EM-2)

RP-HPLC trace at 0 minute:

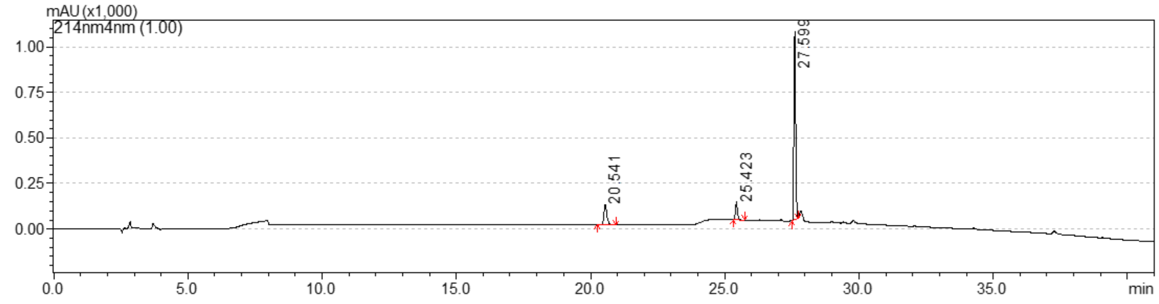

Peak at ^{t}R = 27.599 min; Tyr-Pro-Phe-Phe-NH_2 (EM-2)
Peak at ^{t}R = 20.541 min; internal standard (H-Trp-OH)
Peak at ^{t}R = 25.423 min; internal standard (Z-Lys-OH)

RP-HPLC traces (absorbance at 215 nm), recorded for selected time points (0 minute, 1 h, 2 h and 3 h):

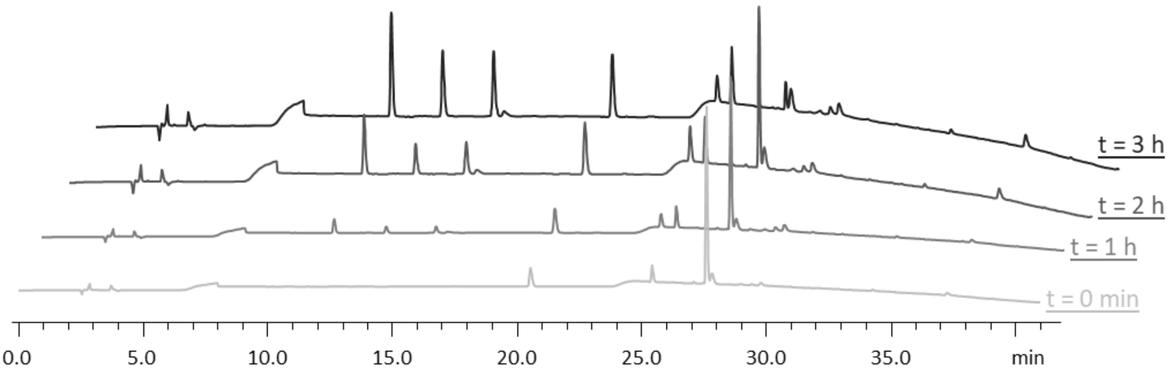

Time-dependent changes in the percentage of the remaining EM-2 subjected to proteolytic degradation in human serum.

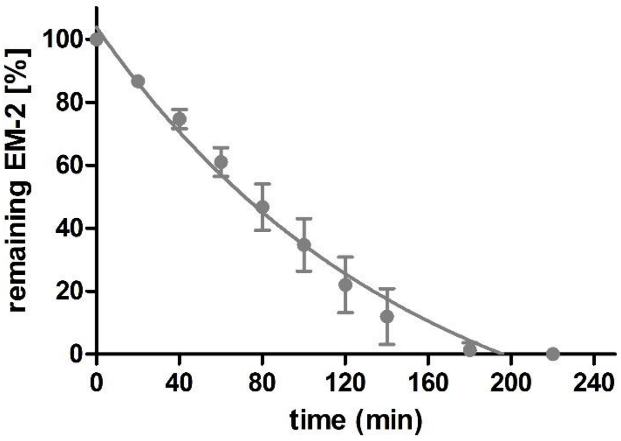

Table SI-4. Selected inhibitors of the VEGF-A_{165}/NRP-1 interaction (or NRP-1 ligands), illustrative for the role of C-terminal Arg residue/fragment.

Entry	Sequence/structure	Activity	Notes	Reference
**A7R analogues**				
**D1**	**A7R** *(parent)*<br>Ala-Thr-Trp-Leu-Pro-Pro-**Arg^7**	82% inhibition at 100 μM	Inhibition of ^{125}I-VEGF binding to recombinant rat NRP-1/Fc chimera	(Starzec et al. 2007)
**D2**	**des-Arg^7-A7R**<br>Ala-Thr-Trp-Leu-Pro-Pro^6	18% inhibition at 100 μM		
**D3**	**[Ala^7]-A7R**<br>Ala-Thr-Trp-Leu-Pro-Pro-**Ala^7**	17% inhibition at 100 μM		
**D4**	**[Lys^7]-A7R**<br>Ala-Thr-Trp-Leu-Pro-Pro-**Lys^7**	20% inhibition at 100 μM		
**D5**	**A7R-Ala^8**<br>Ala-Thr-Trp-Leu-Pro-Pro-Lys^7**-Ala^8**	18% inhibition at 100 μM		
**KPAR tetrapeptides**				
**D6**	Lys-Pro-Ala-**Arg**<br>*(parent)*	83% inhibition at 100 μM, IC_{50} = 30 μM	Inhibition of ^{125}I-VEGF binding to PAE/NRP1 cells	(Jarvis et al. 2010)
**D7**	Lys-Pro-Ala-**D-Arg**	22% inhibition at 100 μM		
**D8**	Lys-Pro-Ala-**Lys**	22% inhibition at 100 μM		
***N*^{α}-substituted arginines**				
**D9**	*N*^{α}-Boc-Arg-OH	K_D = 3 μM	SPR measurements of binding to immobilised NRP1-b1.	(Mota et al. 2018)
**D10**	*N*^{α}-Cbz-Arg-OH	K_D = 17 μM		
**D11**	*N*^{α}-benzoyl-Arg-OH	K_D = 24 μM		

continued next page

Entry	Sequence/structure	Activity	Notes	Reference
**Peptidomimetics**				
**D12**	**EG00229**<br>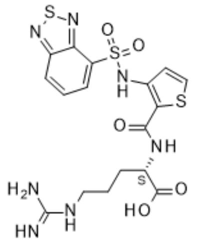	99% inhibition at 100 μM, IC_{50} = 8 μM		
**D13**	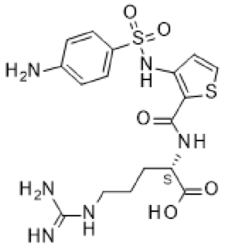	78% inhibition at 100 μM, IC_{50} = 13 μM	Inhibition of ^{125}I-VEGF binding to PAE/NRP1 cells	(Jarvis et al. 2010)
**D14**	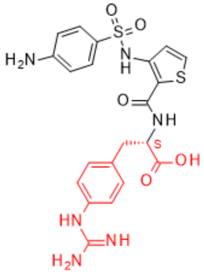	13% inhibition at 100 μM,		

Entry	Sequence/structure	Activity	Notes	Reference
**D15**	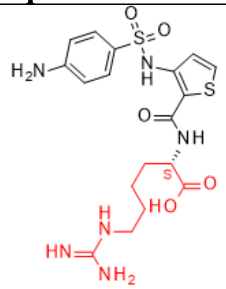	20% inhibition at 100 μM,		

References (only for supplementary information)

Jarvis A, Allerston CK, Jia H, et al (2010) Small molecule inhibitors of the neuropilin-1 vascular endothelial growth factor A (VEGF-A) interaction. J Med Chem 53:2215–26. https://doi.org/10.1021/jm901755g

Mota F, Fotinou C, Rana RR, et al (2018) Architecture and hydration of the arginine-binding site of neuropilin-1. FEBS J 285:1290–1304. https://doi.org/10.1111/febs.14405

Starzec A, Ladam P, Vassy R, et al (2007) Structure–function analysis of the antiangiogenic ATWLPPR peptide inhibiting VEGF165 binding to neuropilin-1 and molecular dynamics simulations of the ATWLPPR/neuropilin-1 complex. Peptides 28:2397–2402. https://doi.org/10.1016/j.peptides.2007.09.013

Trott O, Olson AJ (2010) AutoDock Vina: Improving the speed and accuracy of docking with a new scoring function, efficient optimization, and multithreading. J Comput Chem 31:455–461. https://doi.org/10.1002/jcc.21334
